# A conformational switch high-throughput screening assay and allosteric inhibition of the flavivirus NS2B-NS3 protease

**DOI:** 10.1371/journal.ppat.1006411

**Published:** 2017-05-25

**Authors:** Matthew Brecher, Zhong Li, Binbin Liu, Jing Zhang, Cheri A. Koetzner, Adham Alifarag, Susan A. Jones, Qishan Lin, Laura D. Kramer, Hongmin Li

**Affiliations:** 1 Wadsworth Center, New York State Department of Health, 120 New Scotland Ave, Albany NY, United States of America; 2 Department of food science, College of food science and technology, Guangdong Ocean University, Zhanjiang, Guangdong, People’s Republic of China; 3 Center for Functional Genomics, University at Albany, Rensselaer, New York, United States of America; 4 Department of Biomedical Sciences, School of Public Health, State University of New York, Albany, New York United States of America; NIH, UNITED STATES

## Abstract

The flavivirus genome encodes a single polyprotein precursor requiring multiple cleavages by host and viral proteases in order to produce the individual proteins that constitute an infectious virion. Previous studies have revealed that the NS2B cofactor of the viral NS2B-NS3 heterocomplex protease displays a conformational dynamic between active and inactive states. Here, we developed a conformational switch assay based on split luciferase complementation (SLC) to monitor the conformational change of NS2B and to characterize candidate allosteric inhibitors. Binding of an active-site inhibitor to the protease resulted in a conformational change of NS2B and led to significant SLC enhancement. Mutagenesis of key residues at an allosteric site abolished this induced conformational change and SLC enhancement. We also performed a virtual screen of NCI library compounds to identify allosteric inhibitors, followed by *in vitro* biochemical screening of the resultant candidates. Only three of these compounds, NSC135618, 260594, and 146771, significantly inhibited the protease of Dengue virus 2 (DENV2) *in vitro*, with IC_50_ values of 1.8 μM, 11.4 μM, and 4.8 μM, respectively. Among the three compounds, only NSC135618 significantly suppressed the SLC enhancement triggered by binding of active-site inhibitor in a dose-dependent manner, indicating that it inhibits the conformational change of NS2B. Results from virus titer reduction assays revealed that NSC135618 is a broad spectrum flavivirus protease inhibitor, and can significantly reduce titers of DENV2, Zika virus (ZIKV), West Nile virus (WNV), and Yellow fever virus (YFV) on A549 cells *in vivo*, with EC_50_ values in low micromolar range. In contrast, the cytotoxicity of NSC135618 is only moderate with CC_50_ of 48.8 μM on A549 cells. Moreover, NSC135618 inhibited ZIKV in human placental and neural progenitor cells relevant to ZIKV pathogenesis. Results from binding, kinetics, Western blot, mass spectrometry and mutagenesis experiments unambiguously demonstrated an allosteric mechanism for inhibition of the viral protease by NSC135618.

## Author summary

Many flaviviruses such as Dengue virus (DENV), West Nile virus (WNV), and Zika virus (ZIKV) cause severe human diseases. However, specific therapies do not exist to treat these virus infections. The flaviviruses encode a protease complex composed of viral NS3 protein and an NS2B cofactor which is essential for generation of functional virus particles. Previous studies indicate that the NS2B cofactor displays both active and inactive conformations and that the active conformation can be allosterically inhibited. Here we developed a conformational switch assay that can monitor the conformational transition of NS2B and used this assay to characterize allosteric inhibitors. Using a computational approach, we identified potent allosteric inhibitors for the viral protease. The most potent inhibitor could not only inhibit the protease activities of DENV and ZIKV but also inhibited the growth of flaviviruses including DENV, WNV, ZIKV, and YFV in cells relevant to the disease development. Overall, our results demonstrate that targeting the conformational change of NS2B is a valid approach for therapeutic development, and that our assay is suitable for high throughput screening of large compound libraries to identify novel allosteric inhibitors.

## Introduction

Dengue virus (DENV) and Zika virus (ZIKV), members of the *Flavivirus* genus, are mosquito-borne pathogens responsible for a large disease burden. Over 2.5 billion people are at risk of DENV infections worldwide with approximately 50–100 million cases, 500,000 severe cases, and 22,000 deaths per year [1]. These infections, which in severe cases develop into hemorrhagic fever, primarily occur in tropical and subtropical climates where the DENV vector, the mosquito *Aedes aegypti*, is prevalent. Recently, significant outbreaks of ZIKV, an emerging mosquito-borne flavivirus, have occurred worldwide [2, 3]. ZIKV infections are linked to Guillain-Barré syndrome, as well as an increase in babies born with microcephaly [2, 4–6]. It has been suggested that ZIKV infection during pregnancy can cause severe neurological damage in neonates; WHO has declared ZIKV as a global public health emergency [7]. Although vaccines have been successfully developed for several other flaviviruses, the multiple serotypes of DENV have proven a serious obstacle to vaccine development, leaving large populations at risk [8–11]. Furthermore, due to the dangers and difficulties inherent in mass vaccination of large at-risk populations, it is desirable to be able to treat severe flavivirus infections with antiviral therapeutics that could be administered at the site of an outbreak.

The flavivirus genome is a ~11 kb ss-RNA (+) consisting of one large open reading frame (ORF) encoding a single polyprotein precursor (PP), which is cleaved during and immediately after translation by cellular and a virally encoded protease. These cleavages produce the viral structural proteins: capsid (C), premembrane (prM) or membrane (M), and envelope (E), as well as seven nonstructural proteins required for genome replication: NS1, NS2a, NS2b, NS3, NS4a, NS4B, and NS5 [12]. The NS2b and NS3 proteins together form the viral protease, a trypsin-like serine protease that preferentially cleaves protein substrates at sites immediately following two basic residues [13], including the cleavages between NS2A and NS2B, NS2B and NS3, NS3 and NS4A, and NS4B and NS5; it also makes vital cleavages within the capsid protein (C) [14–18].

The flavivirus NS2B-NS3 protease is a highly conserved and replication-critical enzyme [14, 19–22]. Crystal structures of the NS2B-NS3 proteases of flaviviruses in covalently-linked forms (e.g. NS2B-G_4_SG_4_linker-NS3) have been determined in both apo and inhibitor-bound forms (see reviews [15, 23–25]). In the absence of substrate or active-site inhibitor, the C-terminal portion (Cter) of NS2B adopts an “open” inactive conformation. Upon inhibitor or substrate binding to the NS3 active site, the C-terminal portion of NS2B “wraps around” the NS3 core, closing the NS3 active site with the so-called active “closed” conformation. NS2B conformational changes are required for NS3 function; mutations that abrogate NS2B binding greatly reduce the proteolytic activity of the complex [26, 27]. In addition, the conformational change of NS2B upon active-site inhibitor binding has been verified by a number of NMR studies using the linked construct and by molecular dynamic studies [28–32].

Because these cleavages are essential to viral replication, assembly, and maturation, the NS2B-NS3 protease is an attractive drug target. Most attempts to develop flavivirus protease inhibitors have focused on the NS3 active site with limited success, possibly due to two of the site’s features (see reviews [15, 23–25]). Firstly, the active site is flat and featureless, which makes specific inhibitors unlikely. Secondly, because the active site preferentially binds substrates with basic (positively charged) residues in its P2 and P1 positions, effective inhibitors in biochemical assays are similarly charged; this charge results in poor bioavailability *in vivo*.

Alternate strategies to identify inhibitors that bind not at the NS3 active site, but to another region of the protein to inhibit its function would bypass these difficulties. One such strategy is to allosterically inhibit the viral protease.

In this work, we developed a conformation-switch assay to monitor the conformational change of the viral protease co-factor NS2B. We also performed a virtual screen of the Diversity Set II library of 1,364 compounds from the National Cancer Institute Developmental Therapeutics Program (NCI DTP) against a conformationally dynamic cleft on the face opposite of the protease active site. Functional studies identified one potential inhibitory compound, NSC135618, which inhibited both *in vitro* protease function of DENV2 and ZIKV. NSC135618 was very potent in inhibition of several flaviviruses including DENV, ZIKV, WNV, and YFV, with only moderate cytotoxicity. Overall, our results demonstrate that the conformational change of NS2B is a valid approach for therapeutic development, and our assay is suitable for high throughput screening of large compound libraries to identify novel allosteric inhibitors.

## Results

### Development of a conformational switch assay based on split luciferase complementation (SLC)

It has been shown that upon active-site inhibitor binding to the covalently linked NS2B-NS3, the NS2B C-terminal residues 67–95 undergo dramatic conformational changes to bind the NS3 subunit [15, 33–36]. Our goal was to monitor these conformational changes in a high-throughput manner by exploiting the inducible conformational change of covalently-linked NS2B-NS3 upon active-site inhibitor binding.

To this end, we sought to develop an SLC-based conformational switch assay to monitor the conformational changes of NS2B triggered upon binding of active site-based inhibitor to the NS2B-NS3 protease complex, aiming to identify and characterize allosteric inhibitors that prevent NS2B from forming the active conformation. Notably, SLC has been used to monitor conformational changes previously and to investigate inhibitions of ligand-induced conformational changes [37–43].

**Fig 1:**
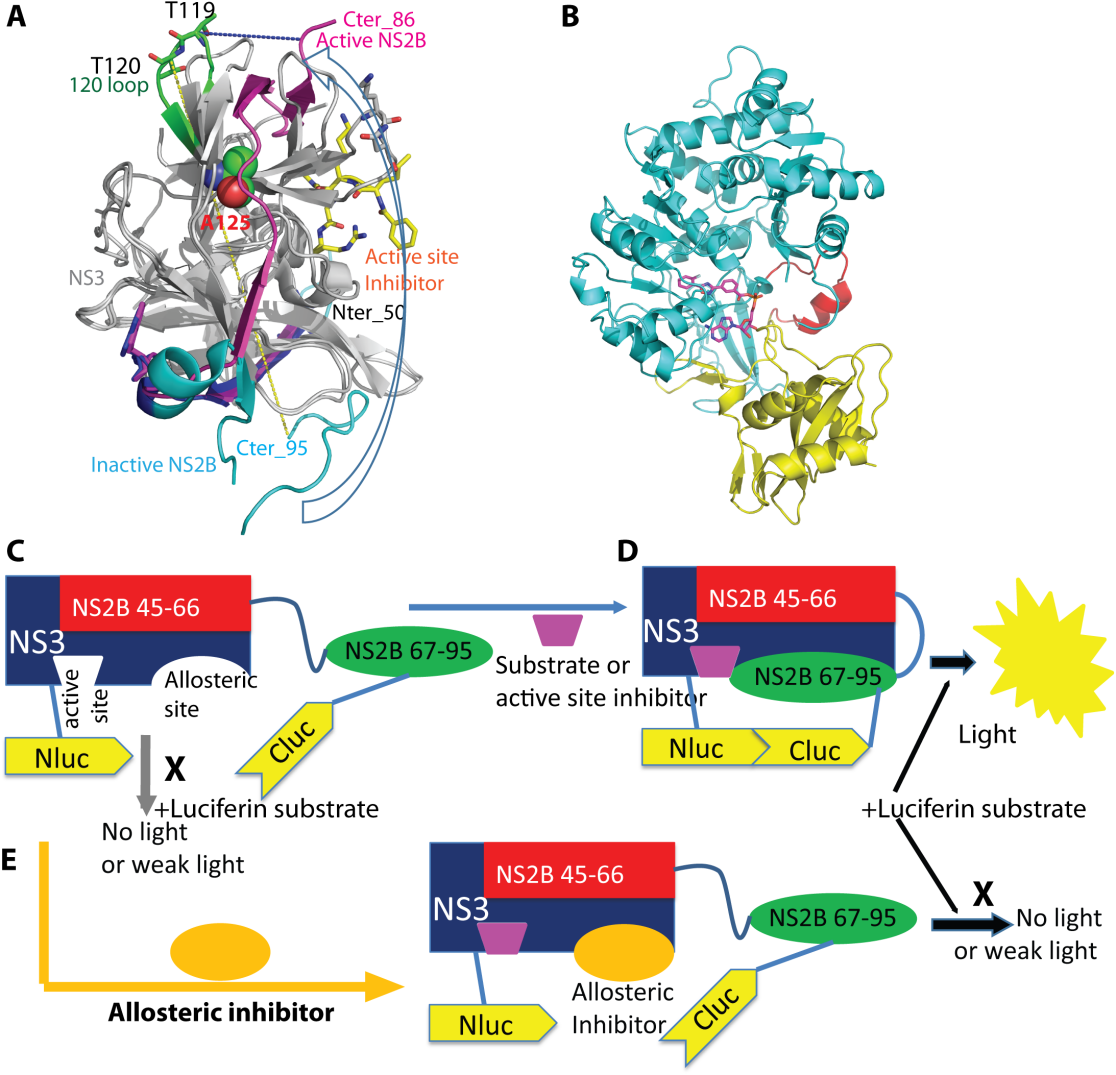
The firefly split luciferase complementation conformational change assay. (**A**) Important features of the active “closed” (PDB: 3U1I, magenta) and inactive “open” (2FOM, cyan and blue) conformations of NS2B. NS3s (gray) of 3U1I and 2FOM were best superimposed. Active-site inhibitor (yellow) and T119 and T120 of the 120 loop of NS3 were in stick representation. N-, C-termini of NS2B, and loops 120 (green) of NS3 were colored and labeled. NS3 residue A125 was in sphere representation. The NS2B N-terminal residues 50–66 of 2FOM were in blue and the C-terminal residues 67–95 of 2FOM were in cyan. Blue arrow indicates conformational change of the NS2B C-terminal portion (Cter) upon active-site inhibitor binding. The distances were in dashed lines: (yellow) between the Cter of NS2B in inactive conformation and the NS3 119 loop: 45Å; (blue) between the Cter of NS2B in active conformation and the NS3 119 loop: 11Å. (**B**) Cartoon representation of firefly luciferase (FLuc) with Nluc (aa. 1–398) in cyan and Cluc (aa. 398–550) in yellow and red. Luciferase inhibitor was shown in stick (magenta). (**C**) Schematic representation of the SLC strategy. When active site is not occupied, NS2B Nter (45–66) remains associated tightly with NS3, whereas NS2B Cter (67–95) is in the “open” conformation. SLC between NLuc and CLuc will not occur. No luminescence will be generated. **(D)** Binding of active-site inhibitor triggers conformational change of the NS2B Cter to form the “closed” conformation. SLC occurs. Luminescent light is generated. **(E)** Binding of allosteric inhibitor to the NS3 allosteric site prevents the NS2B Cter from forming the “closed” conformation, even in the presence of active-site inhibitor. SLC will not occur. No luminescence will be generated.

We noticed that a hairpin loop composed of the NS3 amino acids (aa) 117–122, named as the 120 loop, is close to the NS2B 67–95 hairpin loop in the active conformation (inhibitor bound), but is quite far away from it in the inactive conformation [33] (**Fig 1A**). The distance between the C-terminus of NS2B and the NS3 120 loop is 45Å in the inactive conformation, whereas it is only about 11Å in the active conformation (**Fig 1A**). This feature could be used to develop a conformational switch assay to monitor the conformational change and to identify inhibitors abolishing conformational changes of NS2B.

The principle of this assay is illustrated in **Fig 1B–1E**. Firefly luciferase (FLuc) is composed of 550 amino acids (**Fig 1B**), which can be split into two fragments for the SLC assays: the N-terminal fragment (NLuc) consisting of aa 1–398 and the C-terminal fragment (Cluc) composed of aa 398–550 [44]. As shown in **Fig 1C**, the NLuc fragment can be fused to an NS3 loop such as the 120 loop near the protease active site, whereas the Cluc can be covalently attached to the C-terminus of NS2B. With such a construct, when active-site inhibitor is not present, the C-terminal portion (Cter) of NS2B will not form the “closed” conformation and will not bring the CLuc fragment close to NLuc fused to the NS3 loop (**Fig 1C**). Therefore, SLC will not occur and luminescent light will not be generated (**Fig 1C**). In contrast, when an active-site inhibitor is added, binding of active-site inhibitor triggers a conformational change of NS2B C-terminal fragment, allows it to form the “closed” conformation and brings the CLuc fragment close to NLuc, leading to SLC with a gain-of-signal that can be monitored by a luminometer (**Fig 1D**). Therefore, through monitoring the SLC signal, we can essentially develop an assay to detect the conformational change of NS2B upon active-site inhibitor addition. This assay can be used to screen allosteric inhibitors that prevent NS2B from forming the closed conformation. The principle is shown in **Fig 1E**. If an allosteric inhibitor binds to the allosteric site of NS3, it will block NS2B from forming the closed conformation even in the presence of an active-site inhibitor. Therefore, if a compound can suppress the active-site inhibitor-induced SLC enhancement, that compound will be a potential allosteric inhibitor.

**Fig 2:**
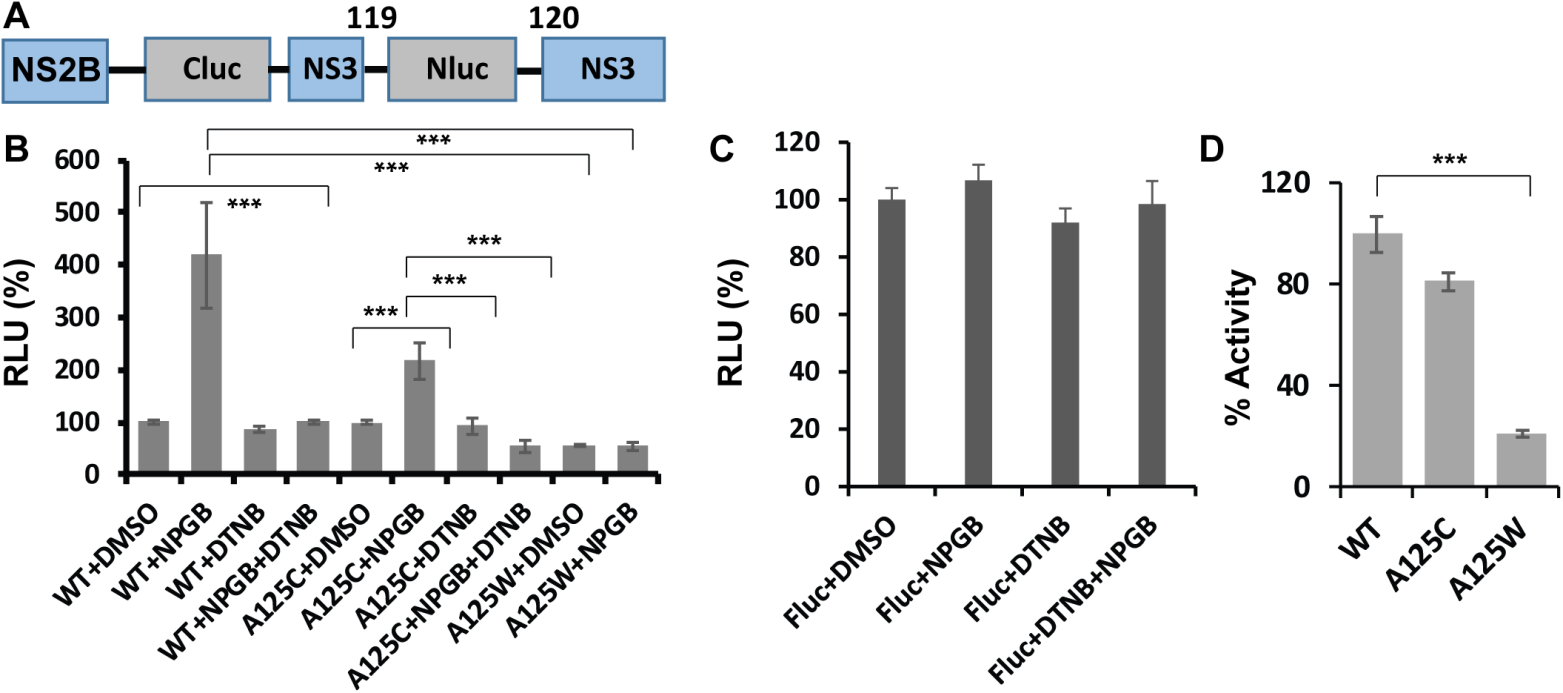
The SLC assays for conformational change of NS2B of DENV2. **(A)** Design of SLC construct for monitoring the NS2B conformational change (based on DENV2). **(B)** Active-site inhibitor NPGB triggers the SLC enhancement that can be suppressed by mutations at an allosteric site A125 of NS3. All proteins were at 150 nM. DTNB and NPGB were at 30 μM. n = 3. ***, p<0.01. (**C**) NPGB and DTNB do not affect the luciferase activity of the full-length luciferase (FLuc) (150 nM). (**D**) Protease activity of the WT and mutant N2CN3N. All proteins (150 nM) were assayed with the Abz substrate (100 μM) for 1 hour. The activity of WT was set as 100%. The relative activities of the mutants were normalized as percentage of the WT activities.

To develop such a conformational switch assay, we generated a covalently-linked construct, NS2B-CLuc-NS3-119-NLuc-NS3 120-to-NS3_C designated as N2CN3N (**Fig 2A**), in which CLuc was inserted between the NS2B C-terminus and the N-terminus of NS3 with a linker GGSGG, and NLuc was fused between NS3 residues 119 and 120 (see their locations in **Fig 1A**).

To evaluate whether the N2CN3N construct can monitor the NS2B conformational changes, we performed the SLC experiment with/without an active-site inhibitor, Nitrophenyl p-guanidinobenzoate (NPGB), which was known to induce conformational change of NS2B by NMR studies [36, 45]. As shown in **Fig 2B**, addition of NPGB (30 μM) significantly increased SLC (4.2-fold), compared to that of the DMSO control. In contrast, NPGB did not have any effects on full-length firefly luciferase (FLuc) (**Fig 2C**). Our results indicated that the active-site inhibitor NPGB likely triggered a conformational change of NS2B from an open conformation to a closed conformation, bringing the NLuc and CLuc fragments close to each other and leading to luciferase complementation and SLC enhancement.

To investigate the assay specificity, we generated two mutant constructs, A125C and A125W, respectively. The NS3 A125 site is a known allosteric site (**Fig 1A**) [46]. It was previously reported that covalent modification of A125C by a thioreactive compound 5,5'-dithio-bis-[2-nitrobenzoic acid] (DTNB) abolished protease activity [46]. Consistent with previous findings [46], the A125C mutation did not significantly change the protease activity (**Fig 2D**). Addition of the active-site inhibitor NPGB to the A12C mutant resulted in SLC enhancement (2.2 fold), which is slightly less than that to the wild-type (WT) (4.2 fold), compared to that of the DMSO control (**Fig 2B**). Addition of the thioreactive DTNB to the A125C mutant only slightly decreased the baseline SLC signal (**Fig 2B**). In contrast, DTNB completely abolished the SLC enhancement of the A125C mutant triggered by active-site inhibitor NPGB (**Fig 2B**). Interestingly, although DTNB did not significantly affect the luciferase activity of the WT N2CN3N construct (**Fig 2B**, WT+DMSO *vs*. WT+DTNB) and the full-length firefly luciferase (**Fig 2C**), DTNB addition significantly abolished the active site inhibitor NPGB-triggered SLC enhancement of the WT N2CN3N construct (**Fig 2B**, WT+DMSO *vs*. WT+NPGB *vs*. WT+NPGB+DTNB). The results may indicate that DTNB inhibits NS2B conformational change through multiple mechanisms, including through reactions with/without the A125C free cysteine. Alternatively, DTNB could interfere with the luciferase complementation instead of interfering the NS2B conformational change. Therefore, the interpretation of this mutation result is not unambiguous.

To further investigate assay specificity and to solve the ambiguity, we attempted to evaluate the effects of permanent modification of the A125 site. We generated an A125W mutant. We first evaluated the mutant effects on the viral protease activity (**Fig 2D**). The A125W mutation significantly reduced the NS2B-NS3 protease activity to about 20% of the WT activity, which is in contrast to A125C (**Fig 2D**). We next evaluated the mutation effects on the SLC conformational change assay. As shown in **Fig 2B**, an A125W mutation reduced the luciferase signal by about 53% in the absence of the active-site inhibitor NPGB, compared to the WT. It is possible that a small portion of NS2B forms transient closed conformation in the absence of active-site inhibitor, leading to baseline luminescence readings in the SLC assay, whereas a W125 mutation from A125 prevents such a small portion of the NS2B C-terminus from forming the “closed” conformation to trigger SLC, and the remaining SLC signal may represent the randomized encounter of NLuc/CLuc in solution. More importantly, the SLC enhancement was not observed when NPGB was added to the A125W mutant, likely because the A125W creates steric hindrance for NS2B to form the “closed” conformation (**Fig 2B**).

Overall, our results indicated that a conformational switch assay was successfully developed to detect the conformational differences with/without active-site inhibitor, which allows the screening and characterization of allosteric inhibitors.

### Identification of candidate allosteric inhibitors by virtual screening

To identify a potential allosteric inhibitor that can be used as a positive control in our assay, we performed virtual screening as an unbiased alternative method other than the assay itself.

**Fig 3:**
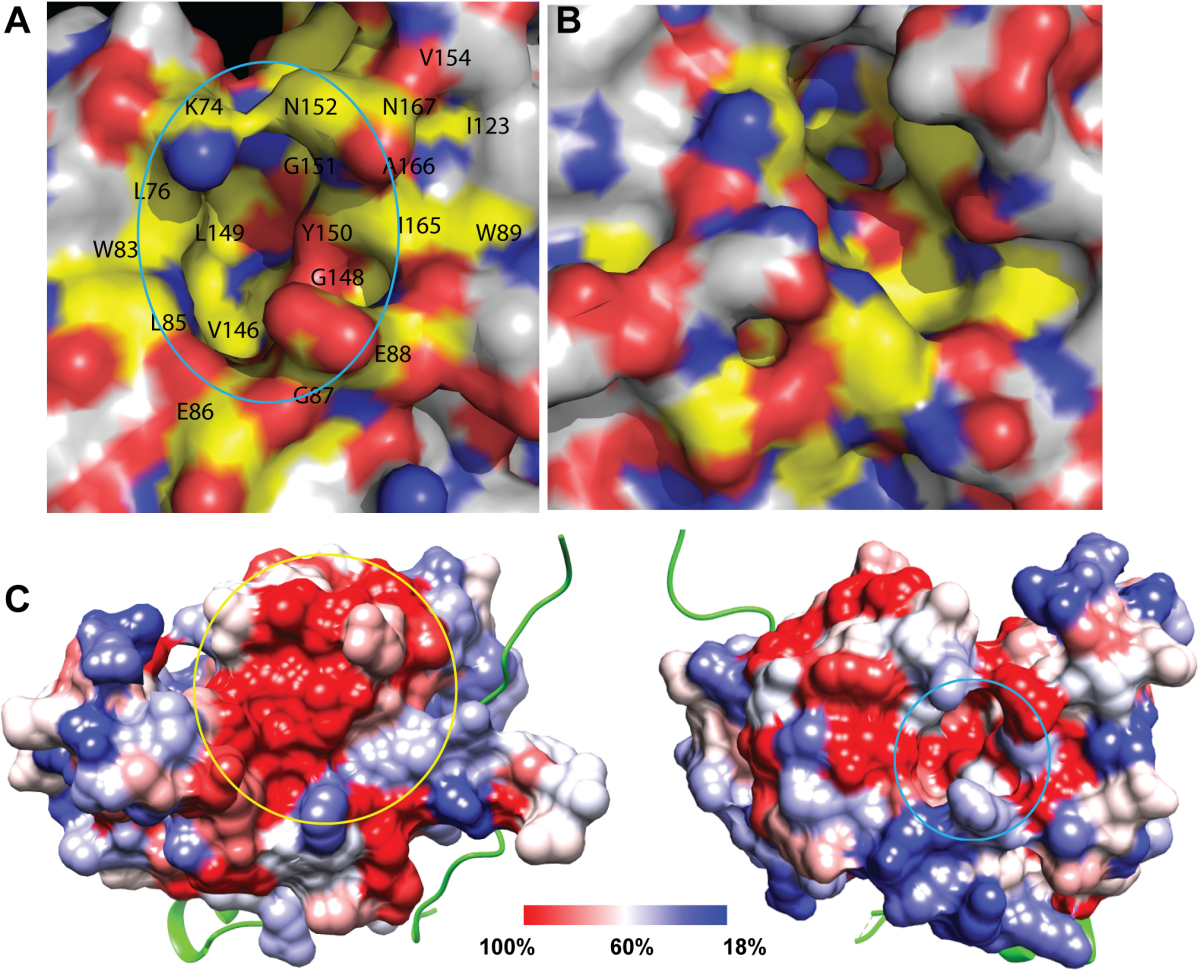
A pocket unique to the inactive NS2B-NS3 structure. **(A)** Surface representation of the unique pocket of the inactive NS2B-NS3 structure. **(B)** Surface representation of the active NS2B-NS3 structure at the same area. Surface color was according to atomic color as follows unless otherwise stated: oxygen, red; nitrogen, blue, carbon of residues involved in pocket formation, yellow; other carbon, white. (C) Mapping of sequence conservation to surface of the DENV2 NS3 protease domain (PDB code: 2FOM); the structure is viewed from two angles, showing features in different regions. Left: active site surface (yellow circle). Right: the unique pocket (cyan circle) of the inactive NS2B-NS3 structure. The surface is colored according to sequence conservation, resulting from the multiple sequence alignment. NS2B is displayed as a cartoon representation (green).

We first compared the crystal structures of the ligand-bound (PDB: 3U1I) and ligand-free (PDB: 2FOM) forms of the DENV protease complexes [19, 33]. A pocket unique to the inactive NS2B-NS3 structure (2FOM) was identified on the NS3 surface opposite to the active site (**Fig 3A**). The pocket is lined up at left side wall by NS3 residues K73, K74, L76 and W83; at right side wall by NS3 residues E88, W89, I123, T118, T120, V147, V154, A164, I165, A166 and N167. The bottom of the pocket was formed by the NS3 residues V146, G148, L149, Y150, G 151 and N152. The pocket was closed at bottom side by the NS3 residues L85, E86, G87 (**Fig 3A**). The same set of residues does not form a pocket on the same surface of the active NS2B-NS3 structure (3U1I), although a pocket with a shape completely different from that of the inactive NS2B-NS3 structure is formed by a different set of residues (**Fig 3B**). Moreover, sequence alignment indicates that the NS3 residues forming the pocket are similarly conserved among flaviviruses as those of active site residues (**Fig 3C**) [15]. Therefore, inhibitors targeting this pocket may potentially be broad spectrum flavivirus inhibitors.

The pocket unique to the inactive conformation as defined above was used to provide a suitable ligand binding site for virtual screening with the program suite of AutoDock Vina [47]. The NCI diversity set II library compounds were docked into the putative allosteric pocket and the 29 top-ranked compounds were selected for further investigation.

**Fig 4:**
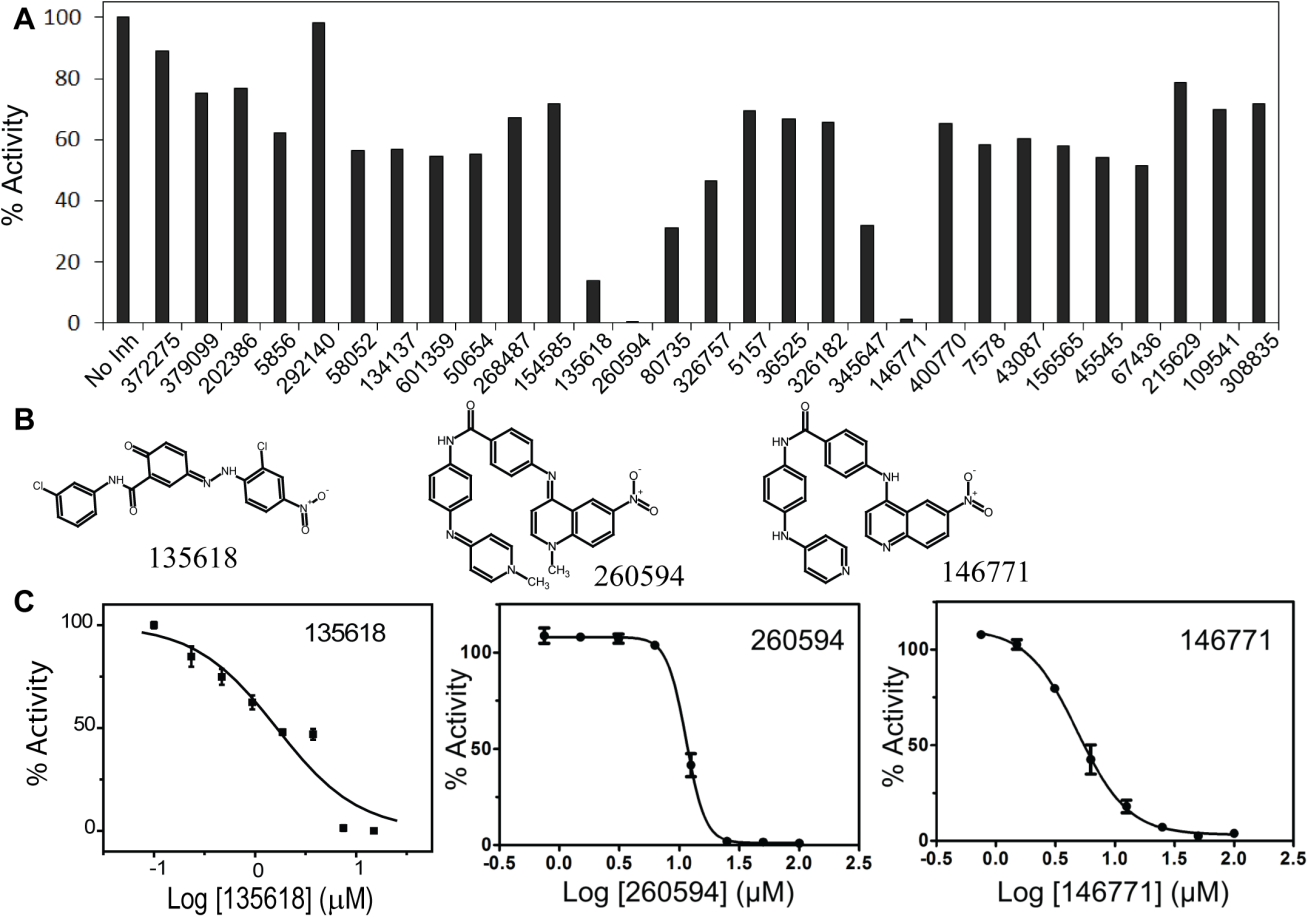
Inhibition of *in vitro* NS2B-NS3pro activity by compounds identified by VS. **(A)** Compounds identified by VS were added to an *in vitro* protease activity assay at a concentration of 100 μM and protease activity relative to the DMSO control was measured. No inhibitor (No Inh) was set as 100%. The protease activities with compounds were set as percentage of the No Inh control. (**B**) Schematic formulas of selected compounds showing *in vitro* anti-protease activities. **(C)** Concentration curves of *in vitro* protease activity inhibition by compounds NSC135618, 20594, and 146771. Compounds were incubated with the linked DENV2 NS2B-NS3 protease (150 nM) for 30 min, prior to addition of the Abz substrate (100 μM). Two fold dilutions of 135618 (from 15 μM to 0.23 μM), 260594 (from 100 μM to 1.6 μM), and 146771 (from 100 μM to 1.6 μM) were used. The protease activity of the DMSO control was set as 100%. The protease activities with compounds were set as percentage of the DMSO control. Data was fitted using the Sigmoidal model within the Origin6.0 software suite.

We then proceeded to screen the 29 compounds identified as potential inhibitors of the DENV protease. We constructed and purified a covalently-linked DENV2 NS2B-NS3 protease complex with a GGGSGGGG linker between NS2B and NS3. Based on a well-established fluorescent assay for flavivirus protease activity [14], we developed a blue-shifted Abz-RRRR↓SAG-3-nitrotyrosine substrate for protease activity assay, where the arrow indicates the cleavage site. In order to overcome interference caused by compound auto-fluorescence, we also developed a red-shifted Tamra-RRRR↓SAG-QXL570 substrate. Only three of the twenty-nine compounds (135618, 260594, and 146771) reduced the activity of DENV2 protease by more than 80% when presented at a concentration of 100 μM (**Fig 4A and 4B**). In order to more precisely determine the *in vitro* inhibitory activity of these compounds against DENV2 protease, we performed the protease activity assay with these compounds across a range of concentrations, resulting in dose-dependent inhibitions by all three compounds (**Fig 4C**). Interestingly, the inhibition curve of compound NSC135618 displayed a shallow slope (Hill coefficient = 0.73) relative to 260594 (Hill coefficient = 5.38), and 146771 (Hill coefficient = 2.03). It is noted that 260594 and 146771 are close analogs (**Fig 4B**). The high hill coefficients for 260594 and 146771 may indicate that these compounds act non-specifically through compound aggregation [48]. Nevertheless, the observed IC_50_ values (the concentration of compound at which the protease activity was 50% inhibited) were 1.8 μM, 11.4 μM, and 4.8 μM for NSC135618, 260594, and 146771, respectively (**Table 1**).

**Table 1:** Inhibition of the DENV2 protease activity.

Compound	IC_50_ (μM)	Hill Coefficient
135618	1.8	0.7
260594	11.4	5.4
146771	4.8	2.0

### Inhibition of conformational change of NS2B

**Fig 5:**
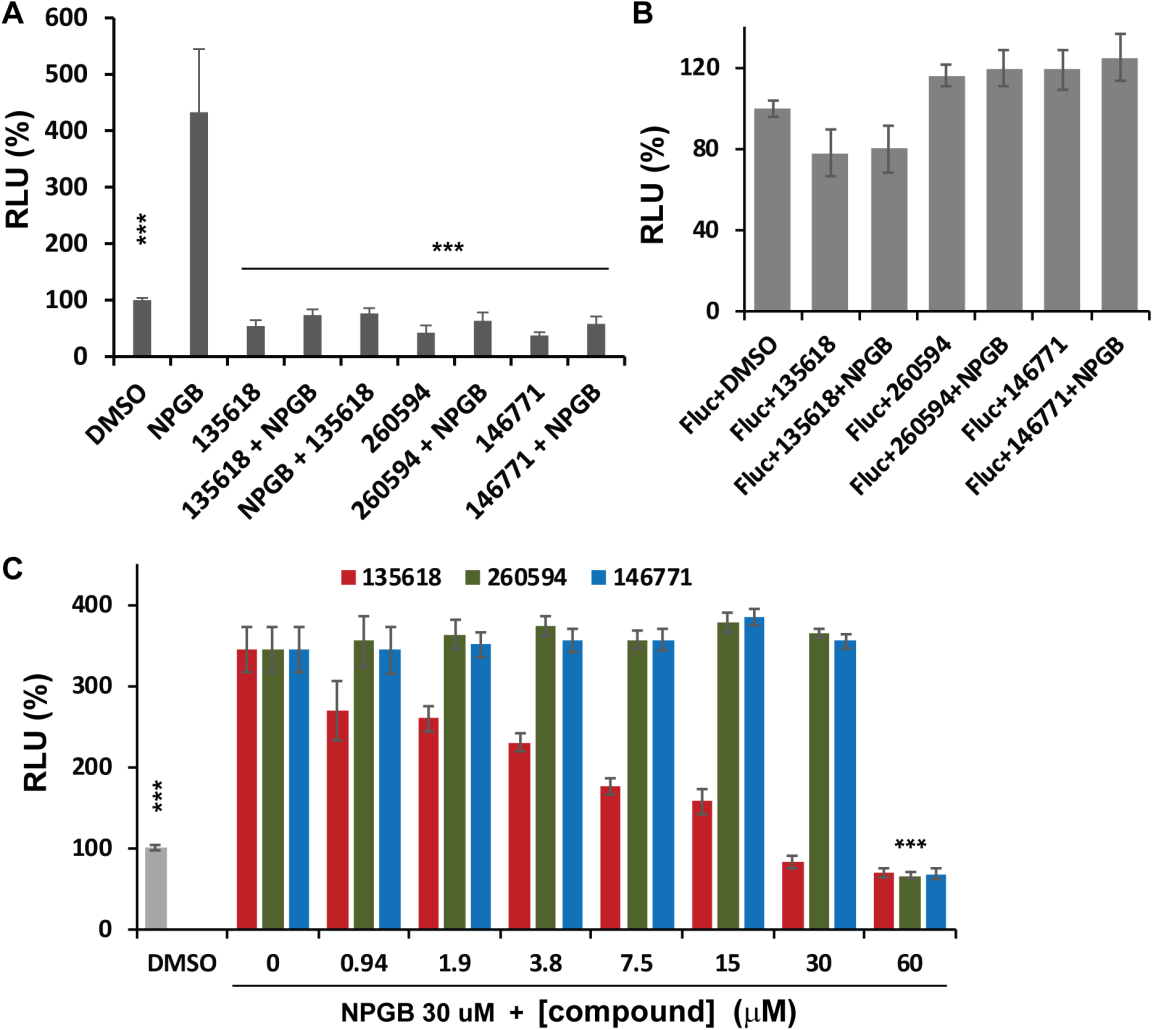
Suppression of NPGB-induced SLC enhancement by candidate allosteric inhibitors. **(A)** Active-site inhibitor NPGB can induce enhanced SLC which can be suppressed by allosteric inhibitors NSC135618, 260594, and 146771 at high concentration. The N2CN3N was at 150 nM. The compounds were at 40 μM. NSC135618 or NPGB was first incubated with the SLC protease construct for 30 min. in different orders, prior to addition of the luciferin substrate, followed by luminescence detection. N = 3. ***, P < 0.01, by one-way ANOVA. (**B**) The candidate compounds do not significantly affect the luciferase activity of FLuc (150 nM). The experimental procedure was the same as above. **(C)** Inhibition of the NPGB-induced SLC enhancement by a concentration series of candidate inhibitors, as indicated. In all panels, DMSO only control was set as 100% relative luminescence (RLU). Luciferase activities of the N2CN3N protease construct with NPGB and/or various compounds were set as percentage of the DMSO control. N = 3. ***, P < 0.01, by one-way ANOVA.

We next evaluated whether the candidate compounds inhibited the conformational change of NS2B induced by active-site inhibitor using the conformational switch assay (**Fig 5**). Addition of candidate allosteric inhibitors NSC135618, 260594, and 146771 at high concentrations (40 μM) appeared to abolish the SLC enhancement induced by NPGB to the background level (DMSO), whereas compounds alone did not have significant effect on background SLC (compared to DMSO) (**Fig 5A**). The order of additions of NSC135618 and NPGB did not make any difference, indicating that the conformational change is reversible (**Fig 5A**, 135618+NPGB *vs*. NPGB+135618). In addition, these compounds did not significantly affect the FLuc activity at high concentration (40 μM) (**Fig 5B**). To further investigate the inhibition mechanism, we performed serial dilution experiment (**Fig 5C**). As shown, compounds 260594 and 146771 did not inhibit the NPGB-induced SLC enhancement until very high concentration (**Fig 5C**). The result is consistent with those of *in vitro* protease inhibition, which show high hill coefficients for the two analogs. The results indicate that the two analogs, 260594 and 146771, may not be true allosteric inhibitors of NS2B conformational changes. In contrast to these two compounds, NSC135618 clearly inhibited the NPGB-induced SLC enhancement in a dose dependent manner (**Fig 5C**). Our results indicated that compound NSC135618 inhibited the conformational change of NS2B triggered by active-site inhibitor and could serve as a positive control in future high throughput screening assays.

### Inhibition of DENV2 by candidate allosteric inhibitors

**Fig 6:**
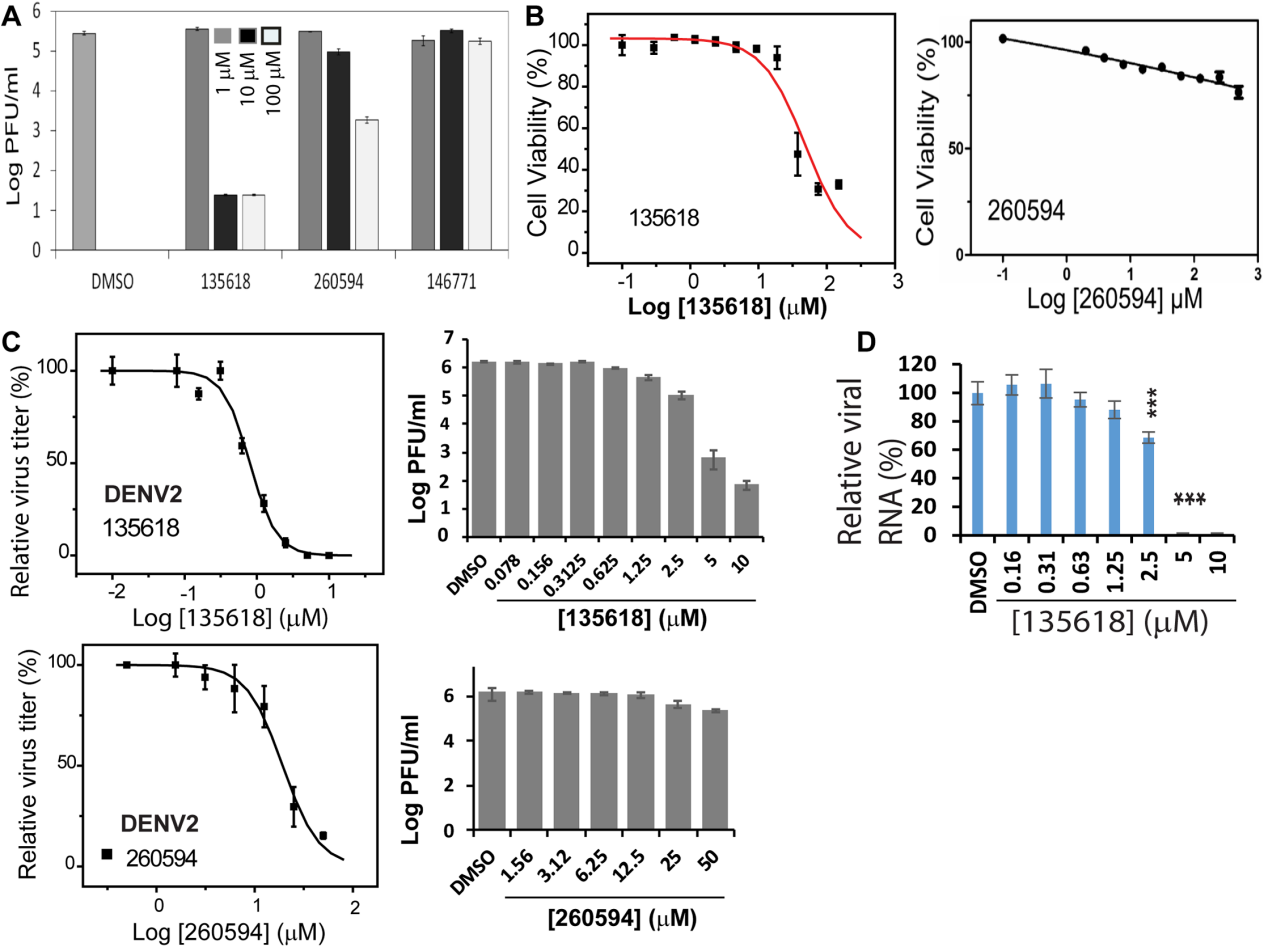
Cell viability and viral reduction activity of candidate compounds in A549 cells. **(A)** Inhibition of DENV2 infectivity on A549 cells by compounds NSC135618, 260594, and 146771 at a concentration of 1 μM (dark grey), 10 μM (black), and 100 μM (light grey) measured as titer of virus produced (on a log scale) relative to the DMSO control. **(B)** Cell viability of A549 cells in the presence of varying concentrations of NSC135618 or 260594. Cell viability for the DMSO control was set as 100%. Cell viability for compound-treated samples were set as percentage of the DMSO control. Curve fitting was performed using the Sigmoidal model within the Origin6.0 software suite. **(C)** Inhibition of DENV2 infectivity by varying concentrations of NSC135618 and 260594. Left: Sigmoidal curve fitting of experimental data expressed as percentage of virus titers (DMSO control was set as 100%). Right: Virus titers at indicated compound concentrations shown in log orders. (**D**) qRT-PCR analysis of inhibition of viral RNA from 135618-treated and DENV2-infected samples in A549 cells. Viral RNA copy number of the DMSO control was set as 100% with those of compounds treated samples as percentage of the DMSO control. For all panels, N = 3. ***, p<0.01, by one-way ANOVA.

In order to determine if these compounds were effective against the replication of actual flavivirus, we infected A549 cells with DENV2 in the presence of several different concentrations of each compound or a DMSO control, and titered the virus produced 48-hours post-infection. We found that viral production was significantly reduced in the presence of compounds NSC135618 and 260594 at concentrations of 10 μM and higher, but that compound 146771 had no appreciable effect **(Fig 6A**). Because it was possible that some of this reduction in viral titer could be the result of compound cytotoxicity, we measured the viability of A549 cells in the presence of a range of compound concentrations using commercially available MTT (for 260594) and WST-8 (for 135618) assays (**Fig 6B**). Compound NSC135618 was moderately toxic towards A549 cells, with a CC_50_ (the concentration of compound at which 50% cells are viable) of 48.8 μM (**Fig 6B**). Conversely, compound 260594 demonstrated very little cytotoxicity on the A549 human lung carcinoma cells even at very high concentrations; a CC_50_ value was not reached, even at 500 μM (**Fig 6B**). Having determined a concentration range at which the A549 cells were viable for each compound, we subsequently performed viral reduction assays to determine the EC_50_ values (the concentration at which viral production is reduced by 50%) for both compounds (**Fig 6C**). The observed EC_50_ for compounds NSC135618 and 260594 were 0.81 μM and 13.5 μM, respectively. In the case of both compounds, the EC_50_ values are well below cytotoxic concentrations, with therapeutic indices of 60 and at least 37, respectively (**Table 2**).

**Table 2:** Cell viabilities and antiviral activities using A549 cells.

Compound	Viruses	EC_50_ (μM)	CC_50_ (μM)	TI^a^
135618	DENV2	0.81	48.8	60
	ZIKV	1.0		49
	WNV	1.27		38
	YFV	0.28		174
260594	DENV2	13.5	>500	37
146771	DENV2	NE^b^	82	N/A

^a^TI: Therapeutic index defined as CC_50_/EC_50_.

^b^NE: Non-effective

Although NSC260594 inhibited the replication of DENV2, its close analog 146771 did not show any inhibition even at very high concentration (100 μM). The antiviral results are not consistent with those of protease inhibition showing that NSC146771 is a more potent protease inhibitor than NSC260594; the antiviral potencies of the analogs are not correlated to the protease inhibition activities. Together with hill coefficient analysis and results from inhibition of the NPGB-induced SLC enhancement, we conclude that NSC260594 and 146771 are not true allosteric inhibitors, and will not be moved forward in further studies

In contrast to NSC260594 and 146771, NSC135618 is an active inhibitor in all assays, including protease inhibition, SLC inhibition, and antiviral efficacy. To further characterize NSC135618, we performed qRT-PCR (**Fig 6D**). Addition of NSC135618 greatly reduced viral RNA copy numbers in a dose dependent manner (**Fig 6D**). Overall, these experiments confirmed that NSC135618 inhibited viral replication, likely because inhibition of the viral protease led to accumulation of unprocessed viral polyprotein precursor (PP), which is replication defective.

### NSC135618 is a broad spectrum allosteric inhibitor

**Fig 7:**
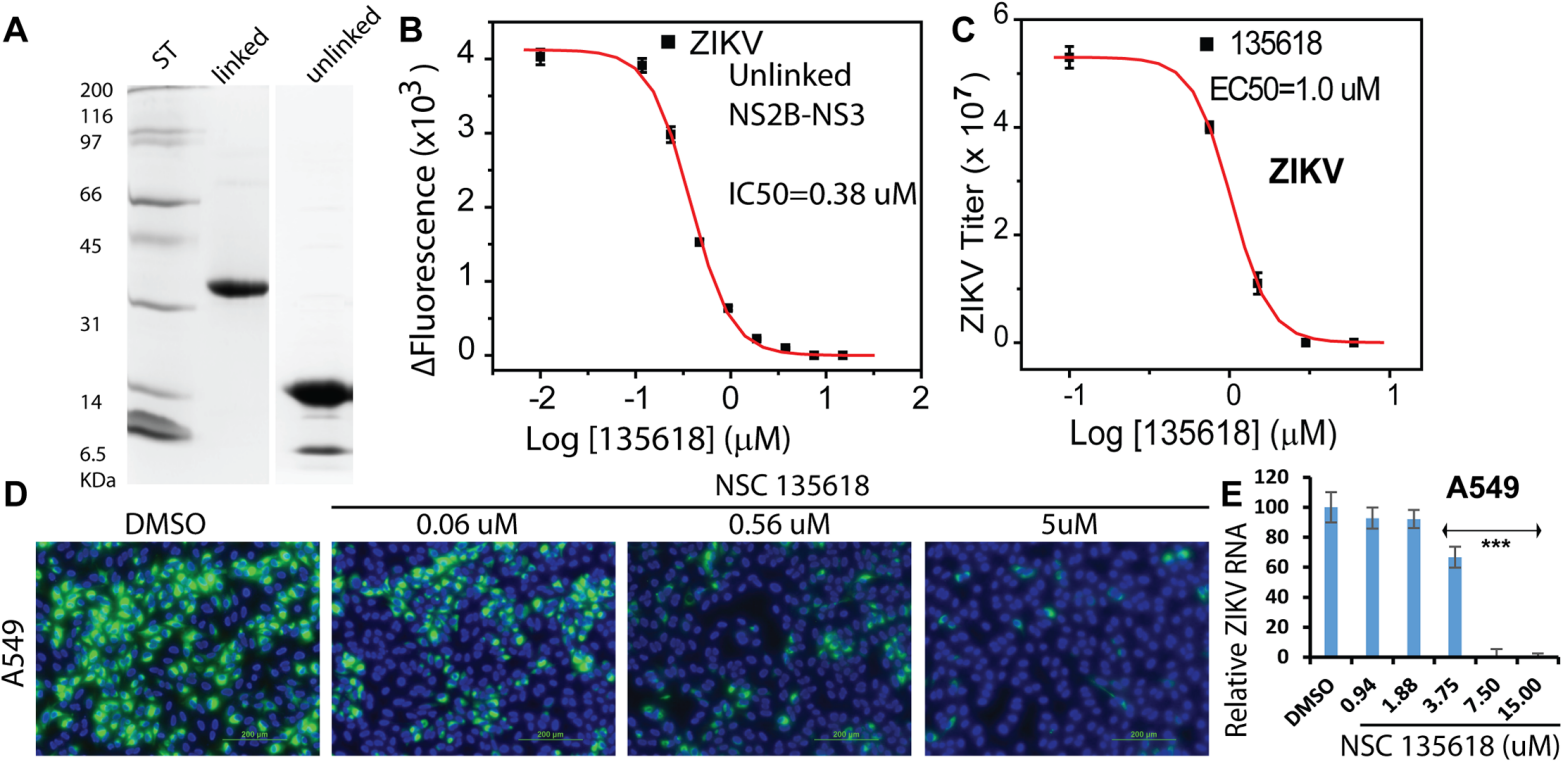
Inhibition of the ZIKV protease activity, viral titer, protein expression, and viral RNA replication by NSC135618. **(A)** SDS-page analysis of purified linked and unlinked NS2B/NS3 protease complex of ZIKV. ST, molecular weight (MW) standard. (**B**) Dose response inhibition of the unlinked ZIKV NS2B-NS3 protease by NSC135618. The NS2b/NS3 protease was at 150 nM. N = 3. (**C**) Dose-dependent inhibition of ZIKV by NSC135618 in A549 cells. Viral plaque reduction assay was used. N = 3. (**D**) Immunofluorescence assay (IFA) of inhibition of viral protein expression for ZIKV-infected A549 cells by NSC135618, using pan flavivirus anti-E antibody 4G2. (**E**) qRT-PCR analyses of inhibition of viral RNA from supernatant of ZIKV infected A549 cells by NSC135618. N = 3, ***, p<0.01, by one-way ANOVA. All dose response curves were fitted with the Sigmoidal model using the Origin6.0 software suite.

As flavivirus NS3 is highly conserved [15] (**Fig 3C**), it is possible that the inhibitors identified are broad spectrum flavivirus inhibitors. We therefore investigated whether NSC135618 is effective against other flaviviruses. Because ZIKV treatment currently has the most urgent need, we evaluated the efficacy of the compound on ZIKV. To this end, we first constructed an unlinked NS2B/NS3 heterocomplex of ZIKV in the pET28a vector by engineering a 3C protease cleavage site within the GGGSGGGG linker region between NS2B and NS3. The NS2B/NS3 protease was first expressed as a His-tagged covalently linked single chain protein in *E coli*. The unlinked ZIKV NS2B/NS3 protease complex was generated by on-column digestion with thrombin and 3C proteases, followed by gel filtration chromatography. Thrombin cleavage led to removal of the His-tag and 3C cleavage led to cleavage of the linker between NS2B and NS3, resulting in unlinked NS2B/NS3 heterocomplex (**Fig 7A**). Results from protease inhibition assay indicated that NSC135618 inhibited the protease activity of the unlinked ZIKV NS2B/NS3 protease in a dose-dependent manner with IC_50_ of 0.38 μM (**Fig 7B**).

We next evaluated the *in vitro* antiviral efficacy of NSC 135618 against the Puerto Rico strain ZIKV PRVABC59. Our results showed that NSC135618 is also a potent inhibitor for ZIKV in A549 cells with EC_50_ of 1.0 μM (**Fig 7C**). Immunofluorescence assay (IFA) and qRT-PCR analyses showed that addition of NSC135618 greatly reduced viral protein expression and viral RNA copy numbers in a dose-dependent manner (**Fig 7D and 7E**). These results indicated that NSC135618 not only reduced viral titer but also reduced viral gene expression and viral RNA replication of ZIKV.

To further investigate the broad spectrum antiviral efficacy of NSC135618, we performed viral reduction assay on two other significant flaviviruses, WNV and YFV (**Table 2**). Our results show that NSC135618 is also a potent inhibitor for WNV and YFV, with EC50s of 1.27 μM and 0.28 μM, respectively. Overall, our results indicate that NSC135618 is a specific and broad spectrum allosteric flavivirus protease inhibitor.

### Inhibition of ZIKV in human placental epithelial cells (HPECs)

**Fig 8:**
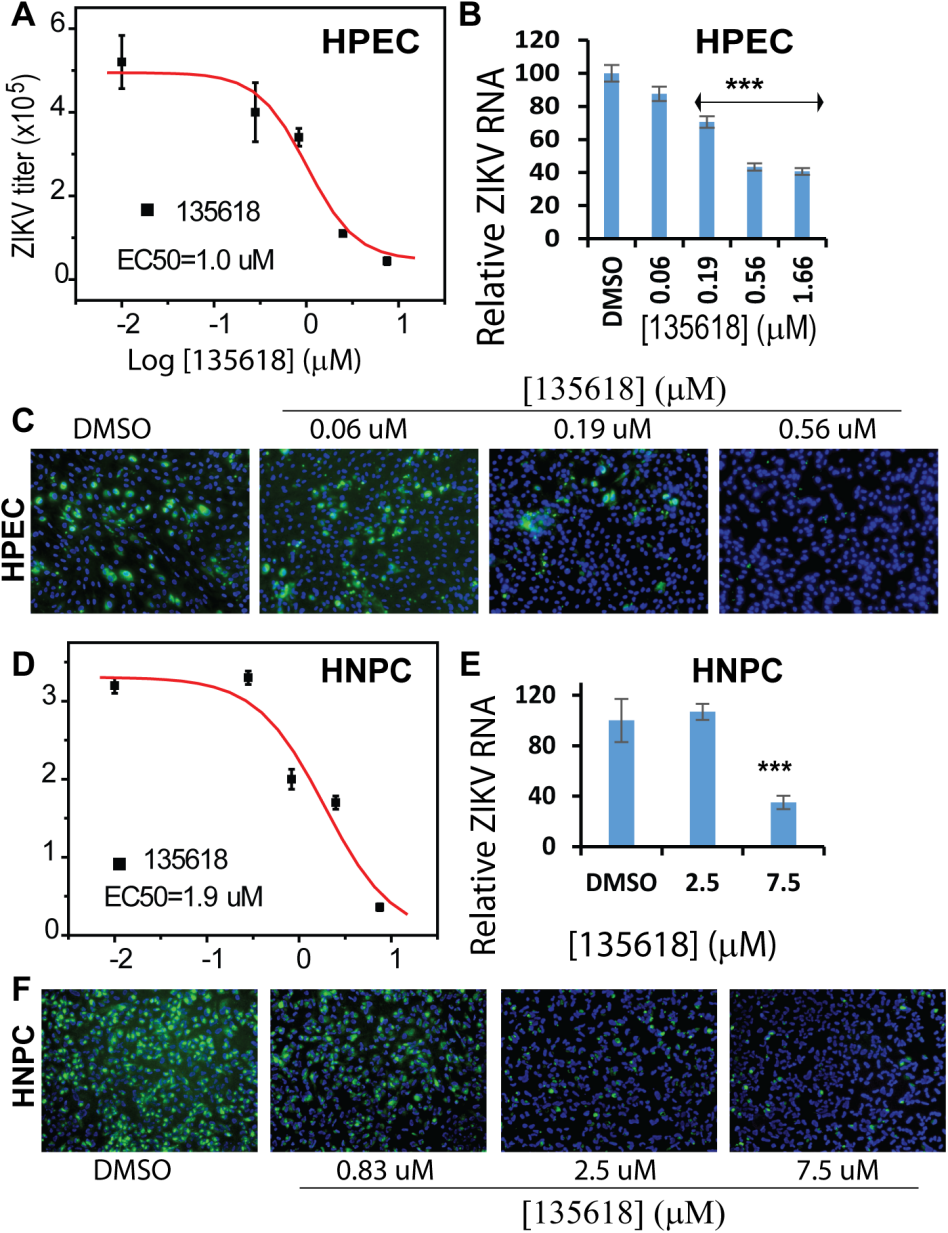
Inhibition of ZIKV in cells relevant to ZIKV by NSC135618. (**A,D**) Dose-dependent inhibition of ZIKV by NSC135618 in human placental epithelia cells (HPECs) (**A**) and human neural progenitor cells (hNPCs) (**D**). (**B,E**) qRT-PCR analyses of inhibition of viral RNA from supernatant of ZIKV infected HPECs (**B**), and hNPCs (**E**) by NSC135618. N = 3. ***, p<0.001, by one-way ANOVA. (**C,F**) IFA of inhibition of viral protein expression for ZIKV-infected HPECs (**C**), and hNPCs (**F**) by NSC135618, using pan flavivirus anti-E antibody 4G2. For all these primary cells, the experiment was performed as described in A549 cells except that ZIKV with MOI of 2 were used. All dose response curves were fitted with the Sigmoidal model using the Origin6.0 software suite.

ZIKV infects fetus and placenta cells during pregnancy, leading to microcephaly in newborns [49]. Therefore, it is valuable to investigate the antiviral efficacy of candidate inhibitors in HPECs. To this end, we used HPECs that are derived from the inner surface of the amnion and have physiological relevance to fetal development and neurogenesis. Our results showed that NSC135618 effectively inhibited ZIKV in HPECs in a dose-dependent manner (**Fig 8A**). The potency for NSC135618 in HPECs is similar to that in A549 cells. In addition, NSC135618 also drastically decreased ZIKV RNA replication and protein expression in HPECs in a dose-dependent manner (**Fig 8B and 8C**). Overall, these experiments demonstrate that NSC135618 is an effective antiviral in placental cells relevant to ZIKV infection.

### Inhibition of ZIKV in human neural progenitor cells (hNPCs)

Because ZIKV also targets hNPCs and neurons [50–53], we further evaluated the drug efficacy in human primary cells related to neurons. To this end, we used hNPCs derived from induced pluripotent stem cell [54]. The results indicate that NSC135618 also considerably reduce ZIKV titers, viral RNA replication, and protein expression in hNPCs in a dose-dependent manner (**Fig 8D–8F**). Overall, these results confirm their likely efficacy for controlling ZIKV infection in these cells.

### Binding of NSC135618 to the NS2B-NS3 protease

**Fig 9:**
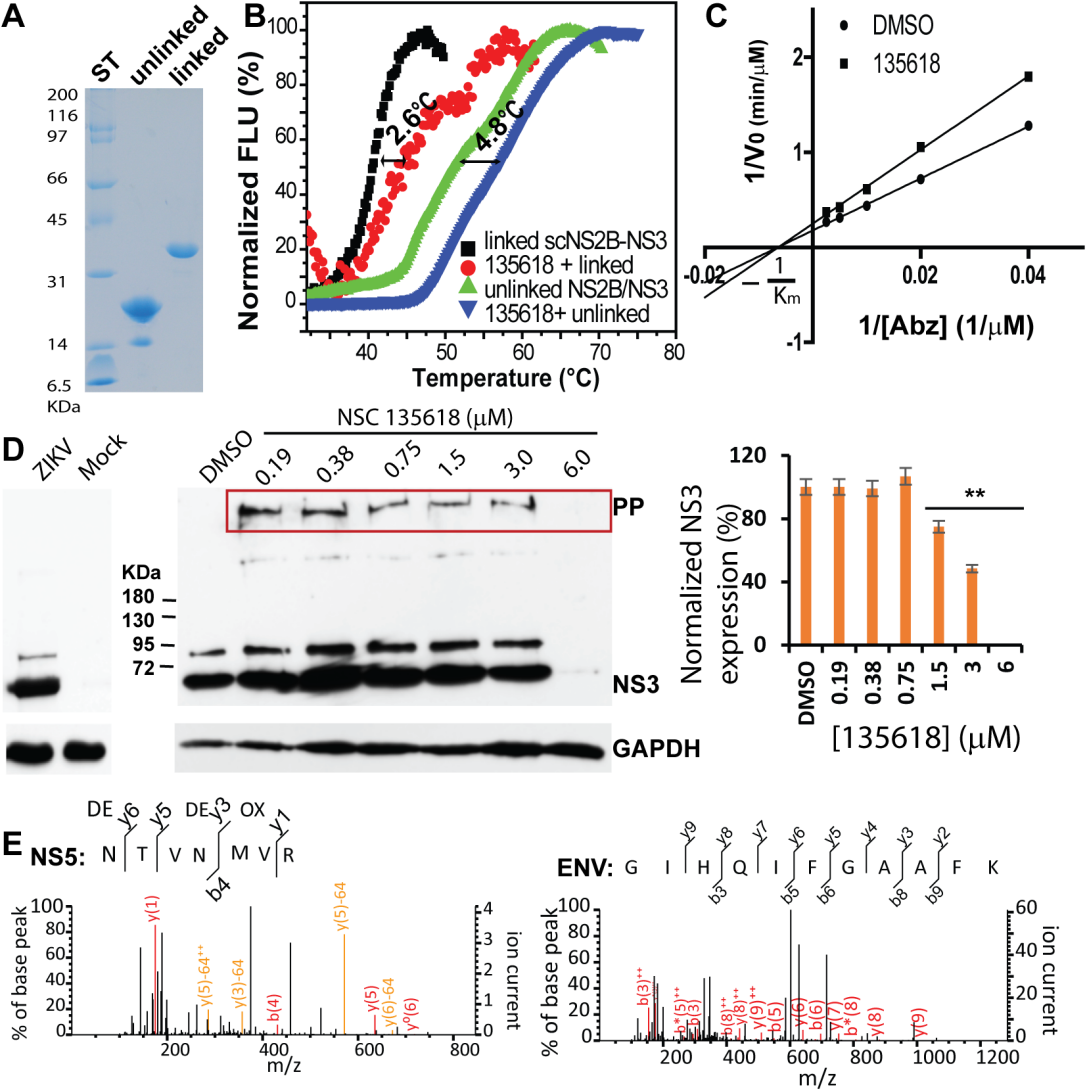
Inhibition of viral polyprotein precursor (PP) processing by NSC135618. **(A)** SDS-PAGE analysis of purified linked and unlinked NS2B/NS3 protease complex of DENV2. **(B)** PTSA for binding of NSC135618 (4.8 μM) to the DENV2 NS2B-NS3 proteins (2.5 μM). Thermal denaturation data was processed using a Derivative model using the Protein Thermal Shift Software v1.0 (ThermalFisher Scientific). ΔT_m_ was defined as T_m-drug_-T_m-DMSO_. N = 3. **(C)** Lineweaver–Burk plot of kinetics experimental data for inhibition of the DENV2 NS2B-NS3 protease complex by NSC135618. The DENV2 NS2B-NS3 (150 nM) was mixed with NSC135618 (3 μM) for 30 min. The Abz substrate was added at various concentrations (400 μM to 25 μM in 2-fold dilutions). N = 3. **(D)** WB analysis of dose-dependent inhibition of ZIKV NS3 expression by NSC135618 using the GTX133309 ZIKV α-NS3 antibody (GeneTex Inc) (left panel). Right panel: NS3 expression (lower bands) normalized to the GAPDH loading control. PP, ZIKV polyprotein precursor. **, p<0.05. **(E)** MS/MS spectra obtained from the fragmentation of the precursor ion at m/z corresponding to representative ZIKV peptides. Fragment ions corresponding to y- and b-ions were observed (red and orange lines).

To determine the mode of action of NSC135618, we performed protein thermal shift assays (PTSA) to investigate binding of the compound to the NS2B-NS3 heterocomplex. We expressed and purified both linked and unlinked NS2B-NS3 proteases of DENV2 (**Fig 9A**). The unlinked DENV2 NS2B/NS3 protease was constructed in a way similar as the ZIKV one, except that thrombin cleavage site was engineered within the linker region. As shown in **Fig 9B**, the protein thermal shift assay (PTSA) [55, 56] demonstrated that NSC135618 binds to both linked and unlinked NS2B/NS3_DENV2_ by increasing their melting temperatures (T_m_) by 2.6°C and 4.8°C, respectively compared to the DMSO control. Compounds with T_m_ change larger than 0.5°C are considered to be protein binders [55, 56]. These data indicate that NSC135618 binds to the NS2B/NS3 protease and stabilizes the protein conformation, leading to *T*_*m*_ increase.

### Non-competitive inhibition of the NS2B-NS3 protease

In order to determine whether or not the compounds inhibited the viral protease by means of an allosteric mechanism as predicted based on virtual screening and conformational switch assay, we measured the enzymatic activity of NS2B-NS3 protease in the presence or absence of inhibitory compounds with varying concentrations of the Abz substrate (**Figs 9C and S1A**). NSC135618 (3 μM) significantly reduced the *V*_*max*_ value for the DENV2 NS2B/NS3 heterocomplex, compared to the DMSO control (**Fig 9C**). Conversely, the *K*_*m*_ values did not change. Similar results were obtained when using the ZIKV NS2B/NS3 protease complex (**S1B and S1C Fig**). These data suggest a non-competitive inhibition mechanism by NSC135618. These results are consistent with a model in which the flavivirus protease activity is reduced by an allosteric mechanism to abrogate its ability to undergo conformational change from an open to closed state.

### Inhibition of viral polyprotein precursor processing

We further used Western blots (WB) to investigate whether the candidate compound could inhibit viral protein expression. Using an anti-ZIKV NS3 antibody, we first verified that the antibody specifically recognized ZIKV NS3, but not proteins from mock infection (no virus) (**Fig 9D**, left panel). We then showed that NSC135618 led to significant inhibition of ZIKV NS3 expression (**Fig 9D**, middle and right panels). Upon normalization to the GAPDH loading control, the ZIKV NS3 expression was inhibited by NSC135618 from 0.75 μM to 6 μM in a dose-dependent manner (**Fig 9D**, right panel). The results are consistent with our hypothesis that allosteric inhibitor such as NSC135618 inhibited viral protease function, leading to reduced expression of viral NS3 protein. At the highest concentration (6 μM), NSC135618 nearly completely suppressed viral protein expression. In addition to the specific NS3 band, an additional band about 80 KDa was frequently shown in some WB. The nature of this band, as illustrated by the vendor’s manual, is currently unknown and could represent an unknown protein non-specifically recognized by the antibody. Alternatively, it could represent the unprocessed NS2B-NS3 complex intermediate.

Nonetheless, for samples treated with NSC135618 at concentrations from 0.19 μM to 3 μM, an accumulation of high molecular weight (MW) protein (>> 180 KDa) was also observed (**Fig 9D**, red boxed in middle panel), which was absent in the DMSO control. The high MW protein could not be detected at the highest drug concentration (6.0 μM).

The high MW product likely represents the unprocessed viral PP (~3,423 amino acids), which accumulates due to inhibition of viral protease by lower concentrations of drugs. For samples treated with inhibitor at high concentration, the absence of PP is likely because of the overall reduced expression of viral protein. Overall, the PP accumulation in the presence of NSC135618 indicated that the PP processing by the viral protease was inhibited. These results are consistent with our hypothesis that inhibitors preventing NS2B conformational change abolish the protease activity, leading to unprocessed PP accumulation.

To further characterize the high MW protein detected, we excised the protein bands, digested with trypsin, and performed mass spectrometry (MS) analysis. Peptides corresponding to the ZIKV envelope, NS3, and NS5 proteins were identified (**Fig 9E**). The mass spectrometry data unambiguously confirmed that the high MW protein was the ZIKV PP.

### N152 is important for binding of NSC135618

**Fig 10:**
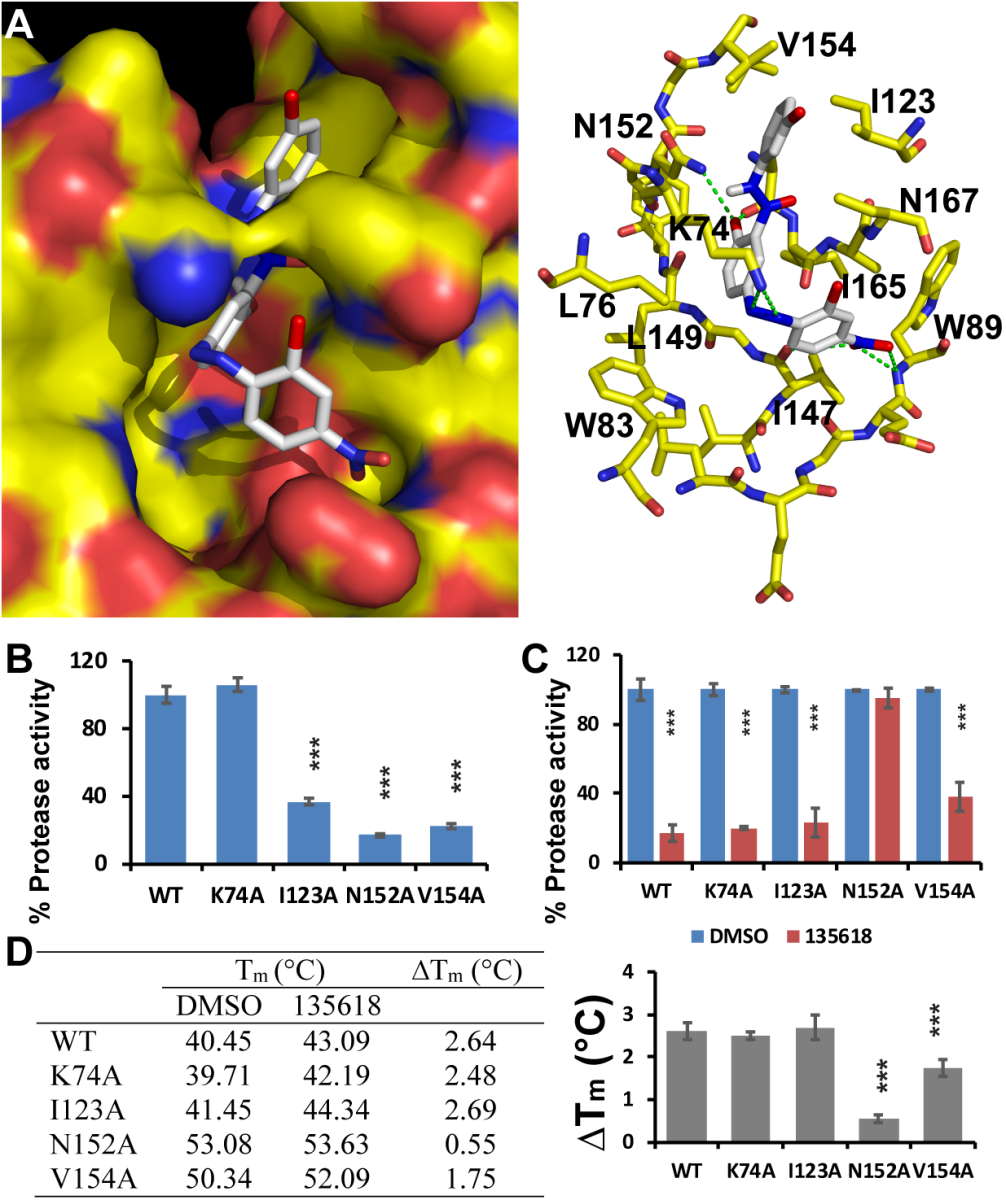
NSC 135618 binding to the unique pocket of the inactive NS2B-NS3 structure. (**A**) Predicted poses of compound NSC135618 in the allosteric pocket of the inactive NS2B-NS3 structure. Left: NS2B and NS3 are in surface representation with surface colors the same as in **Fig 3**. Right: NS3 residues predicted to be in contact with NSC135618 are in stick representation with atomic colors the same as in **Fig 3**. Potential hydrogen bonds are depicted as green dashed lines. NSC135618 is in stick representation with carbons colored white. (**B**) Protease activity of each mutant NS2B-NS3 protein relative to that of WT. All proteins were at 150 nM. The protease activity of WT was set as 100%, with that of the mutant protease as percentage of the WT. N = 3. ***, p<0.01, by one-way ANOVA. (**C**) Relative protease activities of WT and mutant NS2B-NS3 proteins with/without NSC135618 (6 μM). All proteins were at 150 nM. The protease activities of each protein with the DMSO control were set as 100%, with those of compound-treated samples as percentage of the DMSO control. N = 3. ***, p<0.01, by one-way ANOVA. (**D**) PTSA for binding of NSC1351618 (4.8 μM) to the WT or mutant DENV2 NS2B-NS3 proteins (2.5 μM). ΔT_m_ was defined as T_m-drug_-T_m-DMSO_. Data was processed as described above. N = 3. ***, p<0.01, by one-way ANOVA.

The docked conformation of the top inhibitor identified (NSC135618) in the putative allosteric binding site on the DENV2 NS2B-NS3 protease complex was examined to understand the inhibition mechanism (**Fig 10A**). NSC135618 fits into the allosteric pocket with multiple electrostatic and non-polar contacts with the enzyme. There are seven electrostatic contacts between NSC135618 and the NS3 protease, specifically with the sidechains of the NS3 residues Lys74 and Asn152, and the backbone atoms of the NS3 residues Trp89, V147, and A164 (**Fig 10A**). The larger size of this compound allows it to make extensive contacts with as many as 14 amino acid residues from the enzyme (**Fig 10**, right panel).

In order to experimentally determine whether NSC135618 binds into this pocket, we generated several mutants of the DENV2 NS2B/NS3 protease, in which the NS3 residues K74, I123, N152 and V154 were changed to Ala. We first evaluated the protease activity of each mutant (**Fig 10B**). Our data show that except K74A, all other three mutants, I123A, N152A and V154A, greatly reduced the protease activity, with 36%, 17%, and 22% of the WT activity, respectively. Structural analysis indicated that I123, N152 and V154 make contacts with NS2B in the active conformation, whereas K74 does not. We next investigated whether NSC135618 inhibits the protease activity of these mutants (**Fig 10C**). Compared to the DMSO control, NSC135618 retains its inhibition activity for the K74A and I123A mutants. In addition, V154A mutation slightly reduced the inhibition activity of NSC135618. In contrast, N152A mutation completely abolished the inhibition activity of NSC135618 (**Fig 10C**).

We next used PTSA to investigate whether NSC135618 induces T_m_ changes for these mutants. We first noticed that mutations of N152A and V154A significantly stabilized the protein as the T_m_ values for the mutants increased significantly compared to that of WT, whereas K74A and I123A did not have significant impact (**Fig 10D**, left panel). It is currently not clear why these mutations have such a big differential effect on the protease stability. Nonetheless, as shown in **Fig 10D**, K74A and I123A mutations did not alter the T_m_ changes upon binding of NSC135618, compared to WT, suggesting that these mutations did not affect NSC135618 binding. The T_m_ change for V154A is slightly less than that of WT upon NSC135618 binding. Consistently, V154A mutant is less inhibited by NSC135618 than WT. In contrast to other mutations, N152A resulted in almost completely loss of T_m_ change upon NSC135618 binding. The results suggest that N152 is important for binding of NSC135618. The results are consistent with that of docking studies. In the docked model, N152 contributes a hydrogen bond to one hydroxyl group of NSC135618 and makes a number of Van der waals contacts with NSC135618 (**Fig 10A**). Overall these results suggest that NSC135618 binds into the pocket composed of residues N152, and V154.

## Discussion

Crystallography studies indicated that the NS2B C-terminal residues bound to NS3 display large conformational differences between inhibitor-bound and inhibitor-free structures [33–35, 57–61], and even between inhibitor-free structures [33–35, 57–61]. The diversity of conformations of the NS2B C-terminal region indicated that the NS2B C-terminus is highly flexible, and that the conformations seen in the crystal structures may only represent one or a few of many conformations of NS2B in solution. Recently, using unlinked NS2B-NS3 constructs, a few NMR studies indicated that the DENV NS2B-NS3 protease mainly displayed the active “closed” conformation regardless of inhibitor binding [62–65]. However, NMR studies also indicated that (1) the C-terminal portion of NS2B was still found to be flexible; (2) conformational changes were also observed and could be induced upon buffer manipulations; and (3) active-site inhibitor further stabilized the “closed” conformation [36, 63–65]. In addition, recent NMR studies on the ZIKV NS2B-NS3 protease indicated that both closed and open conformations were observed even in the presence of different inhibitors [45, 66]. Overall these data indicated that although it is not clear what exact conformation of NS2B exists *in vivo*, the NS2B C-terminal region is quite flexible and may display multiple conformations, even though the “closed” conformation could be the main species. This flexibility will provide an opportunity to develop small molecule inhibitors to prevent NS2B from forming the correct “closed” conformation, leading to an inactive protease.

Compounds targeting allosteric sites have proven an effective strategy for developing many different therapeutics [67]. For example, efavirenz, navirapene, and delavirdine are non-nucleoside inhibitors that target an allosteric site of HIV reverse transcriptase that locks the enzyme in its inactive conformation [68–70]. HIV protease can also be allosterically inhibited [71]. Similarly, protein tyrosine phosphatase 1B, a type II diabetes target which dephosphorylates insulin receptor, is allosterically inhibited by Benzbromarone and its derivatives [72].

Although allosteric inhibition of drug targets is a well-accepted concept, it is usually difficult to develop assays to monitor allosteric inhibitions in a high throughput manner. It normally requires extensive post kinetic analyses, and often extensive co-crystal structure analyses in order to confirm a true allosteric inhibitor [67]. However, in the case of flavivirus NS2B-NS3, it has been demonstrated that upon active-site inhibitor binding to the linked NS2B-NS3, the NS2B C-terminal residues (aa 67–95) undergo dramatic conformational changes to bind NS3 (**Fig 1**).

In this work, we exploited linked NS2B-NS3's capacity to undergo inducible conformational change when triggered by the binding of an active-site inhibitor. We successfully developed an SLC-based conformational switch assay to monitor the conformational changes of NS2B associated with binding by an active site-based inhibitor. We further showed that the assay was specific to NS2B conformational change, as mutations at an allosteric site known to be essential for NS2B conformational change abolished the luciferase complementation triggered by active-site inhibitor NPGB. Certainly, SLC may have its own limitations, such as identification of false positives not inhibiting the target interactions but instead inhibiting the luciferase itself or inhibiting luciferase complementation. Such false positives could be easily eliminated using luciferase assays with pure full-length luciferase or a SLC-system with irrelevant proteins. Through such a secondary assay, we demonstrated that DTNB is not a specific inhibitor for the NS2B/NS3 protease.

Moreover, we identified a unique pocket present only on the surface of the inactive structure of the DENV2 NS2B-NS3 protease complex, but not on the surface of the active NS2B-NS3 structure. We followed up by virtual screening of the NCI diversity set II small molecule compound library against this unique pocket. Three compounds were found to inhibit the NS2B-NS3 protease activity and one compound, NSC135618, could significantly suppress the conformational change-induced luciferase complementation using our conformational switch assay. Two other compounds, NSC260594 and 146771 which are close homologs, were found to non-specifically inhibit the protease. Search of the PubChem Database indicated that there are about 69 records for NSC 260594 as an active compound in various screening assays, indicating that 260594 is likely a pan assay interference compound [73]. In contrast, compound NSC 135618 was only active in one drug screen, the NCI *in vivo* Anticancer Drug Screen, indicating that NSC135618 is likely a specific allosteric inhibitor of the NS2B-NS3 protease complex.

Results from virus reduction assays indicated that NSC135618 is a broad spectrum flavivirus protease inhibitor with high potency. It could effectively inhibit the growth of DENV2, ZIKV, WNV, and YFV in cell culture. Particularly, NSC135618 displayed sub-micromolar antiviral activity with an *EC*_*50*_ of 280 nM, 810 nM and 1.0 μM for YFV, DENV2 and ZIKV, respectively.

Furthermore, NSC135618 was also found to inhibit the ZIKV protease with sub-micromolar IC_50_ of 0.38 μM. It inhibited ZIKV growth not only in A549 cells but also in human placental and neural progenitor cells relevant to ZIKV pathogenesis.

Finally using the PTSA, WB, SLC conformational switch, kinetics, MS, and mutagenesis approaches, we demonstrated that NSC135618 inhibited viral protease function *via* a non-competitive and allosteric mechanism, leading to accumulation of unprocessed viral polyprotein precursor, and eventually resulting in inhibition of virus growth.

In a summary, we demonstrate that flaviviral proteases can be targeted for allosteric inhibition, and that conformational change is a valid approach for therapeutic development. The successful development of a conformational switch assay provides an opportunity to screen more potent allosteric inhibitors.

## Materials and methods

### Compounds

Compounds were obtained from the NCI DTP Open Chemical Repository (https://dtp.cancer.gov/organization/dscb/obtaining/default.htm). Nitrophenyl p-guanidinobenzoate (NPGB) and 5,5'-dithio-bis-[2-nitrobenzoic acid] (DTNB) were purchased from Sigma-Aldrich.

### Cloning, expression and purification

For cloning of the DENV2 NS2B-NS3 protease complex, an overlapping PCR strategy was used. Viral RNAs were extracted from the culture fluid of Vero cells (American Type Culture Collection (ATCC)) infected with the New Guinea C strain of DENV2, using a QIAamp Viral RNA Mini Kit (Qiagen Inc) according to manufactory’s instructions. The cDNAs of NS2B and NS3 were amplified using the Qiagen OneStep RT-PCR kit (Qiagen Inc) with pairs of primers of 1394F and 1440C for NS2B and 1476F and 1660C for NS3, respectively (**S1 Table**). All the DNA oligoes used in this manuscript were purchased from Integrated DNA Technologies, Inc, unless otherwise specified. The PCR products of the above reactions representing the NS2B residues 48–95 and NS3 residues 1–185 were purified and mixed together as the template to PCR amplify the NS2B_48-95_-G_4_SG_4_-NS3_1-185_, using the primers 1394F and 1660C (**S1 Table**). The obtained PCR product was inserted into the pET28a at the *Nhe I* and *EcoR I* sites.

To create unlinked DENV2 protease, a thrombin cleavage site was engineered into the linker region between NS2B and NS3 using the Strategene point mutagenesis approach, with the following primers (Throm_F and Throm_R) (**S1 Table**).

The ZIKV NS2B-NS3 with a linker containing the 3C protease recognition sequence between NS2B and NS3 was codon optimized and synthesized by BioBasic, Inc, and sub-cloned into the pET28a vector between NdeI and EcoRI sites. The construct contains the NS2B residues 49–95 and the NS3 residues 1–185 of the Puerto Rico strain ZIKV PRVABC59 with a cleavable linker GLEVLFQGPGSG.

The DENV2 NS2B-CLuc-NS3-119-NLuc-NS3 120-to-NS3_C (N2CN3N) construct was cloned into the NS2B-NS3 construct by mega primer PCR mutagenesis approach. First, The CLuc and NLuc fragments, corresponding to FLuc aa 398–550 and 1–398, respectively, was amplified using pairs of primers CLuc_F and CLuc_R, and NluC_F and NLuc_R, respectively (**S1 Table**). The PCR products of the CLuc and NLuc fragments were used as a mega primer for PCR with the DENV2 NS2B-NS3 construct as a template to generate the N2CN3N conformational change construct.

The mutants used in this manuscripts were generated using the Strategene point mutagenesis approach with the primers as shown in **S1 Table**. All constructs were verified by sequencing.

The NS2B-NS3pro proteases of DENV2 and ZIKV as well as the conformational change construct N2CN3N and its mutants were expressed in *Escherichia coli* strain Rosetta 2(DE3) (EMD Biosciences) and purified through a nickel-nitrilotriacetic acid column (Qiagen Inc). The affinity-purified proteases were either directly purified by a gel filtration chromatography or digested using home-made Thrombin and or 3C protease to remove the His-tag, (or cut the linker region), and further purified by a gel filtration 16/60 Superdex 200 column (GE HealthCare).

### Conformational switch assay

The conformational switch assay was carried out in a 96-well plate format. The N2CN3N, the A125C/W mutant protein, or full length Firefly luciferase (150 nM final concentration) was dispensed into test buffer (1x Phosphate buffered saline (PBS), 0.05% Chaps, 0.5% DMSO) with DMSO, DTNB, or candidate compounds at various concentrations, and incubated for 30 min. Then either DMSO or NPGB was added to the mixture to a final concentration of 30 μM. The D-luciferin substrate (Gold Biotechnology, Inc) dissolved in substrate buffer composing of 1x PBS, 2 mM MgCl_2_, 4 mM EGTA, 4 mM ATP, 5 mM DTT was added to a final concentration of 5 μg/ml. The mixture was further incubated for 30 min. The luminescence was recorded with 5 sec integration time using a Veritas microplate luminometer.

### Virtual screening

The program Autodock Vina [47] was used for the molecular docking of the NCI diversity set II library obtained from the https://wiki.nci.nih.gov/display/NCIDTPdata/Compound+Sets web address in January 2011. The sdf format library was converted to PDB format using the program babel [74]. The crystal structure of the inactive form of the DENV2 NS2B-NS3 protease complex (PDB ID: 2FOM) [33] was used as the target protein. A ligand box extending 30 Å in each direction with its center located at a putative allosteric binding site, and an exhaustiveness parameter of 8 was used for the docking.

### Protease inhibition assay

The DENV2 or ZIKV NS2B-NS3pro (150 nM) was mixed with candidate compounds (100 μM, indicated concentration, or DMSO control) in reaction buffer (20 mM Tris pH 8.0, 100 mM NaCl, 5% Glycerol, and 0.05% CHAPS) and incubated at 4^○^C for 30 minutes. The peptide substrate (Abz-RRRRSAG-nTyr (NeoBiolab) or TAMRA-RRRRSAG-GXL570 (AnaSpec)) were first prepared as a stock solution at 50 mM. The peptide was then diluted to the mixture at a concentration 100 μM or 10 μM, respectively, and substrate cleavage was monitored for 1 hour at 37^0^ C in a BioTek Flx800 at excitation/emission wavelengths of 360 nm/420 nm (Abz substrate) or 520 nm/575 nm (TAMRA substrate). The rate of increase in RFU over time was calculated in the linear range and normalized as a percent of the DMSO control.

For all bar graphs and dose responsive curves, all data were presented as means and standard deviations of experimental data in triplicate. Sigmoidal function within the Origin Suite6.0 (Origin Lab, Wellesley Hills, MA) was used for dose responsive curve fittings, unless otherwise specified.

For kinetics analysis, the Abz substrate was used with concentrations from 400 μM to 25 μM in 2-fold dilutions. NSC135618 (3 μM) was incubated with the DENV2 or ZIKV NS2B-NS3 proteases for 30 min prior to addition of the substrate. The rate of increase in RFU over time was calculated in the initial linear range from 0 to 10 min (**S1A and S1B Fig**).

### Cytotoxicity assay

The MTT cell proliferation kit (ATCC) was used to measure the cytotoxicity for compounds 260594 and 146771, according to manufacturer’s protocol and as we described previously [75, 76]. Because the compound NSC135618 interfered with the MTT assay, the cytotoxicity for 135618 was measured by a WST-8 cell proliferation assay (Dojindo Molecular Technologies, Inc), according to manufacturer’s protocol and as we described previously [75, 76].

Briefly, approximately 1 x 10^5^ cells in 100 μl of media were seeded into 60 wells of a 96 well plate, with the remaining wells holding media. Plates were held at RT for 1 hour and then incubated for 20–24 hours in a CO_2_ (5%) incubator at 37°C. The media was removed and 100μl of media containing a 2-fold dilution series of antiviral compound in 1% DMSO were added in triplicate to the wells. After 42-hour incubation at 37°C, MTT or WST-8 assays were performed according to manufactories’ protocols. A microtiter plate reader (Ely808, BioTek Instruments, Inc.) with a 570nm filter (MTT) or 450 nM (WST-8) was used to record absorbance. After adjusting the absorbance for background and comparing to untreated controls, the cytotoxic concentration CC_50_ was calculated using the Sigmoidal nonlinear regression function to fit the dose–response curve using the ORIGIN Suite6.0 (Origin Lab, Wellesley Hills, MA).

### Antiviral assay

A viral titer reduction assay was used to determine the compounds’ effect on selected flaviviruses, including DENV2 (New Guinea C strain), ZIKV (Puerto Rico strain PRVABC59), WNV (NY003356V2 strain), and YFV (17D V3 Vaccine strain) using the A549 lung carcinoma cells (ATCC) as described previously [75, 76]. Briefly, approximately 2 x 10^5^ human A549 cells in 500 μl of media were seeded into a 24 well plate. After seeding for 24 hours, dilutions at 4X the final concentrations of the candidate compound were made in 1% DMSO media and 250 μl was added to wells in triplicate. Then, 250μl of media containing viruses at a concentration to yield a MOI 0.1 PFU/cell was added to the wells. After 48 hours incubation at 37°C, culture media was collected, and stored at -80°C for later quantification using a plaque reduction assay and qRT-PCR. Infected and treated/untreated cells were removed using a cell scraper (Fishersci) and stored at -80°C for Western blot analysis. For the plaque assay, Vero cell monolayers were seeded in 6-well plates 3–4 days prior to infection to achieve a confluent monolayer. Depending on the virus, three to eight, 10-fold serially dilutions of the harvested samples were made. To inoculate, 100 μl of the dilution is inoculated in 6-well plate in duplicate, rocked gently to distribute virus, and incubated for 1 hour at 37°C. Cells are then overlaid with a nutrient medium containing 0.6% oxoid agar. The agar is allowed to solidify and the plates are then incubated at 37°C. A second overlay containing 2% neutral red is added after the plaques begin to appear on day 2, and then incubated overnight. Plaques are counted daily for 1–3 days until no significant increase is seen. The effective concentration EC_50_ was determined by the Sigmoidal nonlinear regression fitting of the dose–response curve using the ORIGIN software package.

Human primary placental epithelial cells (HPECs) derived from the inner surface of the amnion were purchased from Cell Applications, Inc, and cultured according to manufacturer’s manual. Human hNPC, derived from iPSC generated using the STEMCCA Cre-Excisable Constitutive Polycistronic (OKSM) lentivirus, was purchased from EMD Millipore, and cultured according to manufacturer’s manual. The antiviral efficacy experiments with HPECs and hNPCs were carried out as described [75, 76], except that HPECs and hNPCs instead of A549 cells and a MOI of 2 were used.

### Immunofluorescence assay

ZIKV-infected cells treated with DMSO or drugs were grown in 96-well black imaging plates (Corning), similarly as described above in antiviral assay. At 48-hr postinfection, growth medium was removed. The cells were washed once with PBS and fixed on ice in 100% pre-chilled (−20°C) methanol for 15 min. The fixed cells were incubated for 1 hr with blocking and permeabilisation buffer containing 0.5% Triton X-100, 0.2 μg/ml EDTA and 1% BSA in PBS. The cells were then treated with a mouse monoclonal pan anti-E antibody 4G2 (ATCC) overnight and washed three times with PBST buffer (1x PBS with 0.2% Tween 20). The cells were then incubated with the DyLight 488 goat anti-mouse IgG (ImmunoReagents Inc) for 1 hr in blocking buffer, after which the cells were washed three times with PBST. Nuclear staining dye Hoechst was added and incubated for 5 min. Fluorescence images were recorded under a fluorescence microscope equipped with an Olympus DP71 imaging system.

### Quantitative qRT-PCR

50 μl of cell supernatant samples were extracted on Applied Biosystems MagMAX Express-96 Deep Well Magnetic Particle Processor. TaqMan gene expression qRT-PCR assays were performed with 5ul of the extracted RNA using the TaqMan One-step RT-PCR Master Mix Reagents Kit (PE Biosystems) on Applied Biosystems 7500 Real-time PCR System. The TaqMan primers for ZIKV are CCGCTGCCCAACACAAG and CCACTAAYGTTCTTTTGCAGACAT with a ZIKV probe Cy5-AGCCTACCT/TAO/TGACAAGCAGTCAGACACTCAA-IAbRQSp. The TaqMan Primers for DENV2 are CAGGTTATGGCACTGTCACGAT and CCATCTGCAGCAACACCATCTC with a DENV2 probe FAM-CTCTCCGAGAACAGCCCTCGACTTCAA. Samples were analyzed using relative quantification using the 2−ΔΔCT (“delta-delta Ct”) compared with the endogenous control.

### Protein thermal shift assay

The protein thermal-shift assay (PTSA) was conducted using an Applied Biosystem 7500 FAST real Time PCR System (ThermalFisher Scientific) from 25 to 80°C. The linked, unlinked WT or mutant DENV2 NS2B-NS3 (final concentration of 2.5 μM in 1x PBS) was mixed with each compound to attain a 4.8 μM final concentration in 1.6% DMSO in the MicroAmp Fast Optical 96-Well Reaction Plate (ThermalFisher Scientific). Thermal denaturation from 25°C to 80°C was monitored using the SYPRO Orange (Life Technologies) according to manufactory manual. The denaturation of the proteins was monitored by following the increase of the fluorescence emitted by the probe that binds exposed hydrophobic regions of the denatured protein. The melting temperature (T_m_) was calculated as the mid-log of the transition phase from the native to the denatured protein using a Derivative model using the Protein Thermal Shift Software v1.0 (ThermalFisher Scientific). The reference unfolding temperature of proteins in 1.6% DMSO (T_m-DMSO_) was subtracted from the values in the presence of each compounds (T_m-comp_) to obtain thermal shifts, ΔTm = T_m-comp_ ‒T_m-DMSO_. Compounds were considered to be binders when ΔT_m_ > 0.5°C.

### Western blot

Following ZIKV infection at MOI of 0.1, A549 cells treated with DMSO or drugs for 48 hours were washed twice with PBS buffer and manually scraped out the 6-well plates. Cells were spun down and supernatant was removed. 30 μl of complete protease inhibitor cocktail in PBS was added to the cells, followed by addition of 30 μl of SDS-PAGE loading buffer. The mixtures were boiled at 95°C for 10 min, followed by centrifugation at 15,000 rpm for 10 min. Sample was analyzed using 12% SDS-PAGE. The primary antibodies used are anti-ZIKV NS3 (GTX133309, GeneTex, Inc) and anti-GAPDH (CB1001, EMD Millipore), with both at 1:500 dilutions.

### Mass spectrometry

The mass spectrometry experiment was performed at the University of Albany Proteomics & Mass Spec Core facility. The protein band of interest on SDS-PAGE gel was manually excised. The pieces were dehydrated with acetonitrile for 10 min, vacuum dried, rehydrated with 5 mM triphosphine hydrochloride in 50 mM ammonium bicarbonate (pH 8.5) at 37°C for 1 h and then alkylated with 100 mM iodoacetamide in 50 mM ammonium bicarbonate (pH 8.5) at room temperature for 1 h. The pieces were washed twice with 50% acetonitrile, dehydrated with acetonitrile for 10 min, dried, and digested with a total of 25 ng of sequencing grade modified trypsin (Sigma-Aldrich) in 50 mM ammonium bicarbonate (pH 8.5) at 37°C for overnight. Following digestion, tryptic peptides were extracted three times with 50% acetonitrile containing 5% formic acid for 15 min each time while being vortexed. The extracted solutions were pooled and evaporated under vacuum prior to MS analysis.

The peptides were re-suspended in 60 μL of 0.1% vol/vol formic acid and separated on a CapLC system (Waters Co. Milford, MA, USA) coupled to a QSTAR XL (ABSCIEX, Framingham MA). Peptides were desalted onto an Everest C18 (5 μm, 500 μm ID × 15 mm, Grace, Deerfield, IL) with solvent A (97∶3 H_2_O∶ACN with 0.1% vol/vol formic acid and 0.01% vol/vol TFA) at 40 μL/min. After a 6-min wash, peptides were separated on a Jupiter C18 (3 μm, 100 μm ID × 150 mm, Phenomenex, Torrance, CA) using a 40-min linear gradient of 10% to 40% solvent B (85% ACN/10% isopropanol + 0.1% vol/vol formic acid + 0.0075% vol/vol TFA) at 250 nL/min. MS data acquisition was performed using Analyst QS 1.1 software (ABSciex) in positive ion mode for information dependent acquisition (IDA) analysis. The nanospray voltage was 2.1 kV used for all experiments in a positive ion mode. Nitrogen was used as the curtain (value of 20) with heated interface at 130°C. The declustering potential was set at 80 eV and Gas1 was 5 (arbitrary unit). In IDA analysis, after each survey scan from m/z 350 to m/z 1200 and the three highest intensity ions above the predefined threshold 28 cps with multiple charge states (+2 and +3) were selected for tandem MS (MS/MS) with rolling collision energy applied for detected ions based on different charge states and m/z values. Each MS/MS acquisition will be completed and switch back to survey scan when the precursor intensity fell below a predefined threshold or after a maximum of 65 s acquisition. After data acquisition, the individual MS/MS spectrum acquired for each of the precursor within a single LC run were combined, smoothed, deisotoped using an Analyst "script" mascot.dll to create a peak list, the peak list was saved to a file. Then the peak list file was used to query viral protein sub database and contaminations using the MASCOT 2.5 from Matrix Science (London, UK) with the following parameters: peptide mass tolerance, 0.3 Da; MS/MS ion mass tolerance, 0.3 Da; allow up to two missed cleavage; Several variable modifications were applied including methionine oxidation and cysteine carbamidomethylation. Only significant scores for the peptides defined by Mascot probability analysis (http://www.matrixscience.com/help/scoring_help.html#PBM) greater than “identity” with 95% confidence were considered for the peptide identification.

### Statistical analysis

All experiments were performed in triplicates unless specified otherwise. Western blots were quantified using the Bio-Rad Gel Doc EZ system and Image Lab software #1709690 (Bio-Rad). One-way ANOVA was used to carry out statistical analyses with the Prism software.

## Supporting information

S1 Table Primers used. (DOCX)

S1 Fig Kinetics experiment. **(A)** Time course kinetic experiment of the DENV2 scNS2B-NS3 heterocomplex in the presence and absence of NSC135618. The DENV2 scNS2B-NS3 (150 nM) was mixed with NSC135618 (3 μM) for 30 min. The Abz substrate was added at various concentrations (400 μM to 25 μM in 2-fold dilutions). Representative experiments with 400 μM Abz substrate were shown. RFU, relative fluorescence unit. **(B)** Time course kinetic experiment of the linked ZIKV NS2B-NS3 heterocomplex. Experiment condition was the same as in **(A)**. Representative experiments with 400 μM Abz substrate were shown. **(C)** Lineweaver–Burk plot of kinetics experimental data for inhibition of the linked ZIKV NS2B-NS3 protease complex by NSC135618. N = 3.
(TIF)
