# Supplementary material for: A conformational switch high-throughput screening assay and allosteric inhibition of the flavivirus NS2B-NS3 protease
Source: PLoS Pathog. 2017 May 25;13(5):e1006411. doi: 10.1371/journal.ppat.1006411 (PMC5462475; doi:10.1371/journal.ppat.1006411)
Supplement: S1 Table — (DOCX) [file ppat.1006411.s001.docx]

Table S1. Primers used

|  |  | |
| --- | --- | --- |
| 1394F* | | GCGGATCCgctagcGCCGATTTGGAACTGGAGA |
| 1440C | | atactccagc*CCCACCGCCGCCGCTACCACCGCCACC*cagtgtttgttcttcctcttcgt |
| 1476F | | GCGGTGGGGCTGGAGTATTGTGGGATGTCCC |
| 1660C | | GCTCGAGTgcggccgcGAATTCTCActttcgaaaaatatcatcttcgatct |
| Throm_F | | GAGGAAGAACAAACACTG*ggtctggtcccacgtggatcc*GCTGGAGTATTGTGGGATGTC |
| Throm_R | | GACATCCCACAATACTCCAGC*ggatccacgtgggaccag*accCAGTGTTTGTTCTTCCTC |
| Cluc_F^ | | cgaagaggaagaacaaacactg*ggttcc*tccggttatgtaaacaatccggaag |
| Clu_R | | *ccgccgctaccaccgcc*cttggcctttatgaggatctctctg |
| Nluc_F | | caaaacctggtcttttcaaaaccaac*ggcagcggt*GAAGACGCCAAAAACATAAAGAAAG |
| Nluc_R | | gatacggcacctatggttccggc*gccactgcc*CATAATCATAGGACCTCTCACACACAG |
| A125C-F | | GCGCCGGAACCATAGGTTGCGTATCTCTGGACTTTTC |
| A125C-R | | GAAAAGTCCAGAGATACGCAACCTATGGTTCCGGCGC |
| A125W-F | | GCGCCGGAACCATAGGTTGgGTATCTCTGGACTTTTC |
| A125W-R | | GAAAAGTCCAGAGATACcCAACCTATGGTTCCGGCGC |
| K74A-F | | CATGGGCGGACGTTAAGGCGGACCTAATATCATATG |
| K74A-R | | CATATGATATTAGGTCCGCCTTAACGTCCGCCCATG |
| I123A-F | | CAACGCCGGAACCGCGGGTGCCGTATCTC |
| I123A-R | | GAGATACGGCACCCGCGGTTCCGGCGTTG |
| N152A-F | | GTTGTGGGTCTTTATGGTGCGGGTGTTGTTACAAGGAG |
| N152A-R | | CTCCTTGTAACAACACCCGCACCATAAAGACCCACAAC |
| V154A-F | | CTTTATGGTAATGGTGCGGTTACAAGGAGTGGAGC |
| V154A-R | | GCTCCACTCCTTGTAACCGCACCATTACCATAAAG |

*restriction sites underlined, and the linkers in italic fond.

^NS2B: capital letter and underlined; NS3: lowercase letter and underlined
