# Supplementary figures and images for: A conformational switch high-throughput screening assay and allosteric inhibition of the flavivirus NS2B-NS3 protease
Source: PLoS Pathog. 2017 May 25;13(5):e1006411. doi: 10.1371/journal.ppat.1006411 (PMC5462475; doi:10.1371/journal.ppat.1006411)

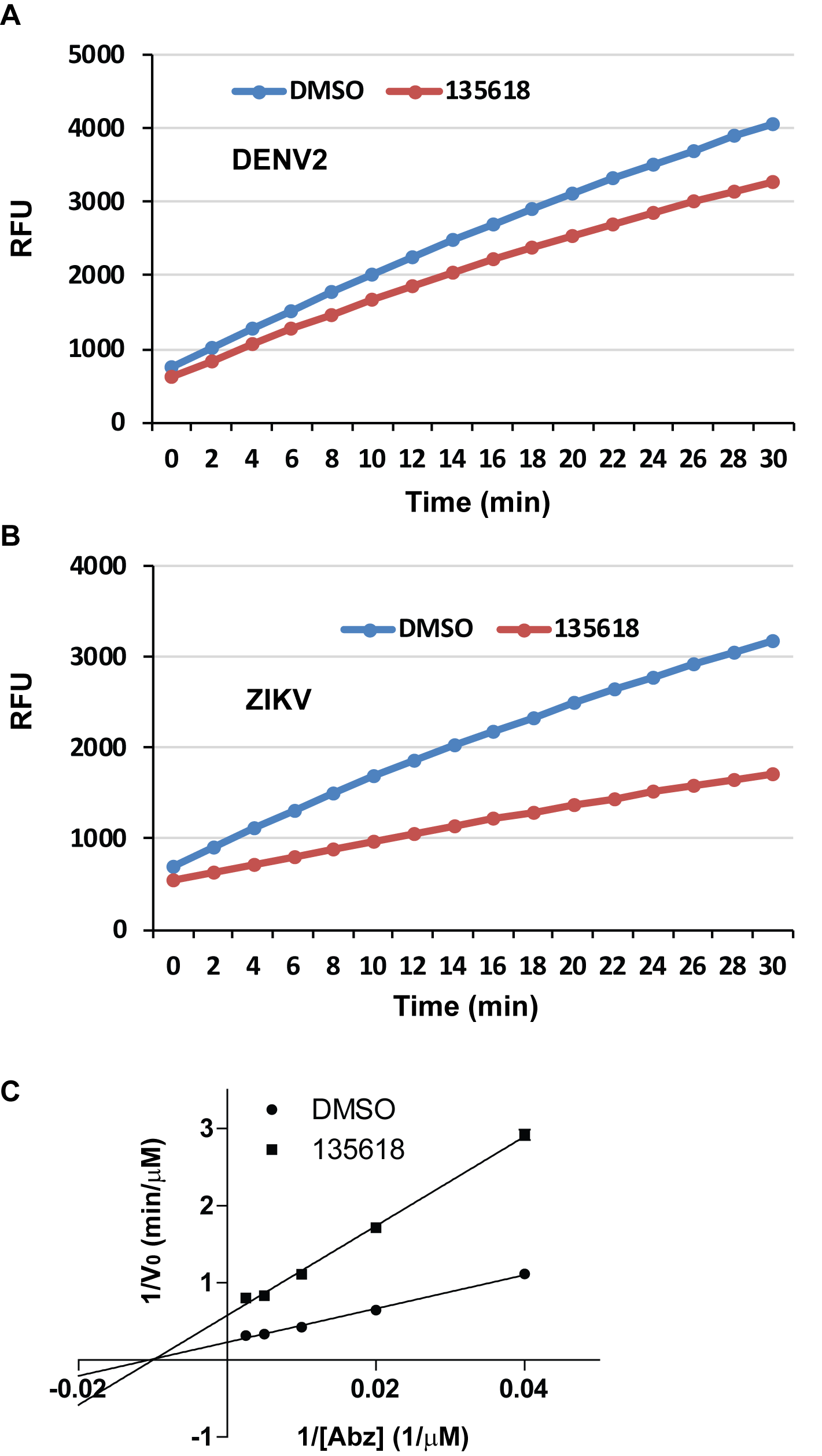

Supplement: S1 Fig — (A) Time course kinetic experiment of the DENV2 scNS2B-NS3 heterocomplex in the presence and absence of NSC135618. The DENV2 scNS2B-NS3 (150 nM) was mixed with NSC135618 (3 μM) for 30 min. The Abz substrate was added at various concentrations (400 μM to 25 μM in 2-fold dilutions). Representative experiments with 400 μM Abz substrate were shown. RFU, relative fluorescence unit. (B) Time course kinetic experiment of the linked ZIKV NS2B-NS3 heterocomplex. Experiment condition was the same as in (A). Representative experiments with 400 μM Abz substrate were shown. (C) Lineweaver–Burk plot of kinetics experimental data for inhibition of the linked ZIKV NS2B-NS3 protease complex by NSC135618. N = 3. (TIF) [file ppat.1006411.s002.tif]
